# Acetyl-CoA flux from the cytosol to the ER regulates engagement and quality of the secretory pathway

**DOI:** 10.1038/s41598-021-81447-6

**Published:** 2021-01-21

**Authors:** Inca A. Dieterich, Yusi Cui, Megan M. Braun, Alexis J. Lawton, Nicklaus H. Robinson, Jennifer L. Peotter, Qing Yu, Jason C. Casler, Benjamin S. Glick, Anjon Audhya, John M. Denu, Lingjun Li, Luigi Puglielli

**Affiliations:** 1 Department of Medicine, University of Wisconsin-Madison, Madison, WI USA; 2 Waisman Center, University of Wisconsin-Madison, Madison, WI USA; 3 Neuroscience Training Program, University of Wisconsin-Madison, Madison, WI USA; 4 School of Pharmacy and Department of Chemistry, University of Wisconsin-Madison, Madison, WI USA; 5 Department of Biomolecular Chemistry, University of Wisconsin-Madison, Madison, WI USA; 6 Wisconsin Institute for Discovery, University of Wisconsin-Madison, Madison, WI USA; 7 Department of Molecular Genetics and Cell Biology, The University of Chicago, Chicago, IL USA; 8 Geriatric Research Education Clinical Center, Veterans Affairs Medical Center, Madison, WI USA; 9 Department of Neuroscience, University of Wisconsin-Madison, Madison, WI USA; 10 Present Address: Harvard Medical School, Boston, MA USA

**Keywords:** Biochemistry, Cell biology, Neuroscience, Diseases

## Abstract

Nε-lysine acetylation in the ER is an essential component of the quality control machinery. ER acetylation is ensured by a membrane transporter, AT-1/SLC33A1, which translocates cytosolic acetyl-CoA into the ER lumen, and two acetyltransferases, ATase1 and ATase2, which acetylate nascent polypeptides within the ER lumen. Dysfunctional AT-1, as caused by gene mutation or duplication events, results in severe disease phenotypes. Here, we used two models of AT-1 dysregulation to investigate dynamics of the secretory pathway: AT-1 sTg, a model of systemic AT-1 overexpression, and AT-1^S113R/+^, a model of AT-1 haploinsufficiency. The animals displayed reorganization of the ER, ERGIC, and Golgi apparatus. In particular, AT-1 sTg animals displayed a marked delay in Golgi-to-plasma membrane protein trafficking, significant alterations in Golgi-based N-glycan modification, and a marked expansion of the lysosomal network. Collectively our results indicate that AT-1 is essential to maintain proper organization and engagement of the secretory pathway.

## Introduction

Nε-lysine acetylation in the Endoplasmic Reticulum (ER) has emerged as an essential component of the quality control (QC) machinery that maintains protein homeostasis (proteostasis) within the ER^1–6^. ER acetylation requires an ER membrane transporter, AT-1/SLC33A1, which translocates acetyl-CoA from the cytosol to the ER lumen, and two ER membrane-bound acetyltransferases, ATase1/NAT8B and ATase2/NAT8, which acetylate ER cargo proteins within the lumen of the organelle^1,3,7^. The acetylation of ER cargo nascent glycoproteins requires ATase1 and ATase2 to interact with the oligosaccharyl tranferase complex (OST) and acetylate correctly folded nascent glycoproteins that are transiting across the ER membrane^1,2^. Studies performed with type I glycoproteins suggest that successful acetylation of the nascent polypeptide is necessary for successful engagement of the secretory pathway^2,8^. Supporting evidence comes from mouse models of increased or decreased ER acetylation^4–6^.

Loss-of-function mutations or gene duplication events in *AT-1/SLC33A1* are associated with severe disease phenotypes spanning from spastic paraplegia (heterozygous mutation) to developmental delay and premature death (homozygous mutations)^9–11^ and intellectual disability with autistic-like traits and progeria-like dysmorphic features (gene duplication)^12,13^. These human disorders have been effectively recapitulated in mouse models. AT-1^S113R/S113R^ mice, which lack AT-1 activity, die during embryogenesis, while AT-1^S113R/+^ mice, a model of AT-1 haploinsufficiency, develop peripheral and central neuropathy as well as propensity to infections and cancer^4^. Mice with neuronal-specific overexpression of AT-1 (AT-1 nTg) develop an autistic-like phenotype^5^ while mice with systemic overexpression (AT-1 sTg) develop a progeria-like phenotype^6^.

In this study, we investigated the outcomes of dysregulated cytosol-to-ER acetyl-CoA flux within AT-1 sTg and AT-1^S113R/+^ mice on the engagement and functional organization of the secretory pathway, as well as the overall quality of secreted glycoproteins (referred to as the secretome). We found that AT-1 sTg animals display enlarged cisternae within the rough ER, enlarged ER Golgi Intermediate Compartment (ERGIC), and a marked delay in protein trafficking to the cell surface, while AT-1^S113R/+^ mice display contraction of the Golgi apparatus. The AT-1 sTg cellular phenotype was associated with significant alterations in Golgi-based N-glycan modification, highlighting changes in the overall quality of the secretome, together with marked expansion of the lysosomal network. Collectively our results indicate that AT-1 is essential to maintain proper organization and engagement of the secretory pathway. Our results also highlight the possibility that the Golgi apparatus harbors a check point for QC of efficient post-translational N-glycosylation, allowing glycoproteins that fail QC to be diverted to the lysosomal compartment.

## Results

### Aberrant AT-1 activity affects secretory pathway-related processes

**Figure 1:**
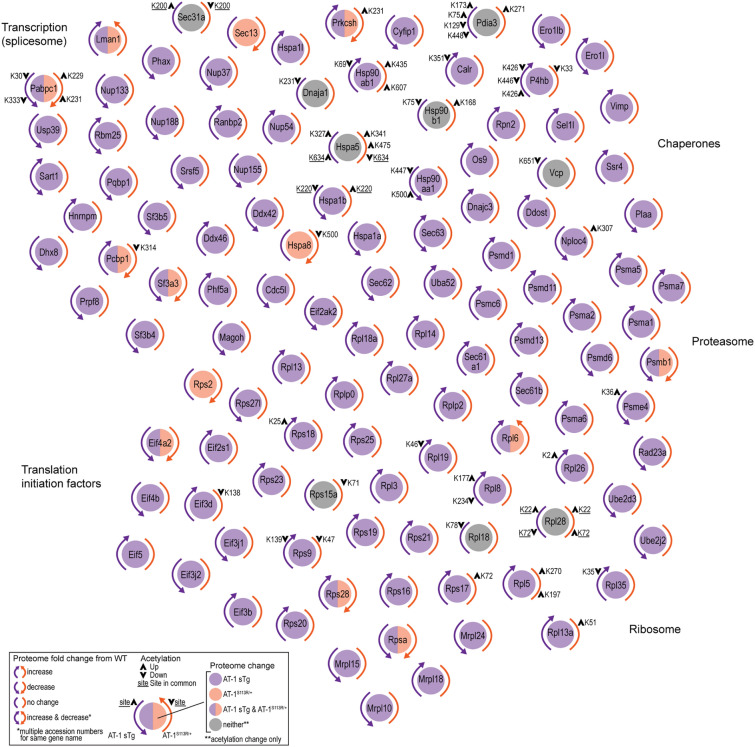
AT-1 sTg and AT-1^S113R/+^ display proteomic changes across secretory pathway-related processes. Proteins with significantly changing abundance and acetylation stoichiometry identified across secretory pathway-related KEGG pathways from liver fractions are shown in clusters (n = 4 WT; n = 4 AT-1 sTg; n = 4 AT-1^S113R/+^). Changes in proteome are indicated by a purple (AT-1 sTg), orange (AT-1^S113R/+^), or grey (neither proteome changed) circle; corresponding colored arrows indicate increase, decrease, or no change in proteome compared with WT. Acetylation stoichiometry changes are designated with a black arrow on the corresponding AT-1 model in which it changed significantly. If a site changes significantly in both models, the site is underlined.

To dissect how changes in cytosol-to-ER flux of acetyl-CoA might affect the secretory pathway, we used mass spectrometry-based strategies^14^ to examine the proteome and acetyl-proteome (stoichiometry of lysine acetylation) of AT-1 sTg and AT-1^S113R/+^ mice. The effect of these genetic manipulations to the cytosol-to-ER flux of acetyl-CoA is reported elsewhere^4,6,14,15^. The analysis identified dynamic changes to protein abundance and acetylation across several essential and functional components of the secretory pathway (Fig. 1, Supplementary Table S1). Comparison of all proteomic datasets indicates that the changes imparted by AT-1 affect the secretory pathway more dramatically than other metabolic pathways^14^. Protein abundance changes found to be enriched in KEGG pathways at either level of regulation (proteome or acetyl-proteome) and in either model were used as the input for clustering. Therewithin, five clear sub-clusters emerged: transcription (spliceosome), translation initiation factors, ribosome, proteasome, and chaperones. Of the 117 proteins found to be enriched, 30 proteins showed significant changes in acetylation and 109 displayed significant changes in overall levels. Finally, 22 proteins showed changes at the level of both the proteome and the acetyl-proteome. The majority of changes imparted in acetylation stoichiometry were observed in two sub-clusters: ribosomes, with 12 proteins, and chaperones, with 10 proteins. Overwhelmingly, 106 of the 117 secretory pathway proteins showed changes in the AT-1 sTg proteome, indicating a robust response to increased cytosol-to-ER acetyl-CoA flux. Of note, we found significant changes within the Sec61, Sec62 and Sec63 complex, which allows translocation of newly synthesized proteins into the ER^16^; Pdia3, Ero1l, Ero1lb and Calr, which ensure formation of disulfide bonds and folding of nascent glycoproteins within the calreticulin/calnexin cycle^17^; and Sec13 and Sec31, which make the outer layer of the coat that captures newly synthesized ER cargo proteins within COPII structures^18,19^.

When taken together, these results indicate that acetyl-CoA homeostasis differentially impacts sub-clusters within the secretory pathway at the level of the proteome, acetyl-proteome, or both. However, in contrast to mitochondria- and lipid-related metabolic clusters^14^, the secretory pathway is largely modulated at the level of the proteome.

### Altered AT-1 activity results in morphological reorganization of the ER, ERGIC, and Golgi apparatus

**Figure 2:**
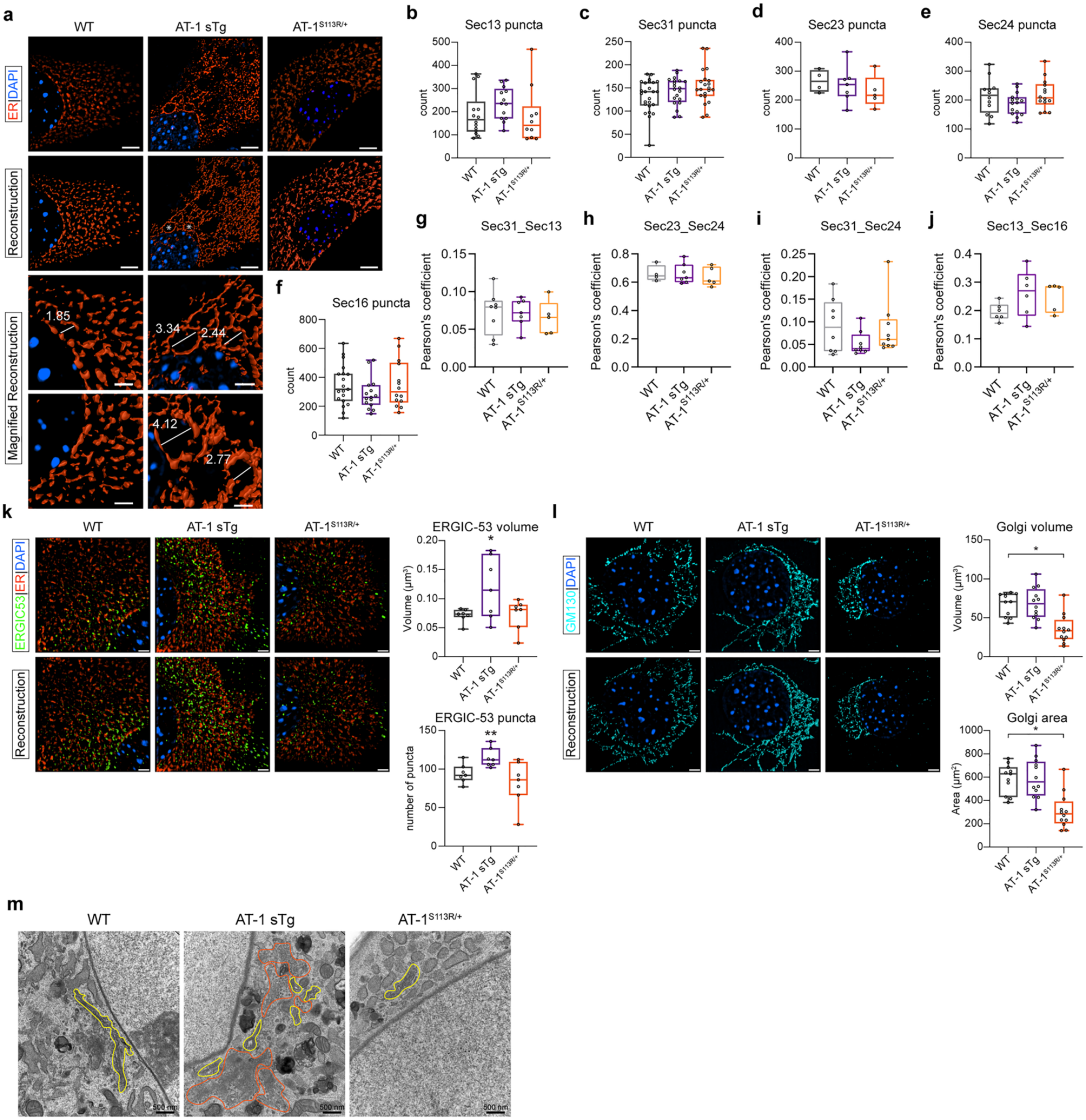
Morphological reorganization of ER, ERGIC and Golgi apparatus in aberrant AT-1 models. (**a**) Representative ER morphology in primary-cultured MEFs using ER3-mCherry (scale bar, 3 µm). White asterisk (*) indicates an enlarged cisternae within the perinuclear rough ER. High-magnification areas for WT and AT-1 sTg are shown (scale bar, 2 µm). (**b**–**f**) Quantification of puncta revealed using SIM microscopy in primary-cultured MEFs in (**b**) Sec13 puncta (n = 14 WT; n = 13 AT-1 sTg; n = 10 AT-1^S113R/+^), (**c**) Sec31 puncta (n = 25 WT; n = 20 AT-1 sTg; n = 21 AT-1^S113R/+^), (**d**) Sec23 puncta (n = 4 WT; n = 7 AT-1 sTg; n = 5 AT-1^S113R/+^), (**e**) Sec24 puncta (n = 12 WT; n = 15 AT-1 sTg; n = 14 AT-1^S113R/+^), (**f**) Sec16 puncta (n = 19 WT; n = 15 AT-1 sTg; n = 14 AT-1^S113R/+^). (**g**–**j**) Pearson’s coefficient of puncta revealed using SIM microscopy in primary-cultured MEFs in (**g**) Sec31 and Sec13 puncta (n = 8 WT; n = 7 AT-1 sTg; n = 5 AT-1^S113R/+^), (**h**) Sec23 and Sec24 puncta (n = 4 WT; n = 7 AT-1 sTg; n = 5 AT-1^S113R/+^), (**i**) Sec31 and Sec24 puncta (n = 8 WT; n = 8 AT-1 sTg; n = 9 AT-1^S113R/+^), (**j**) Sec13 and Sec16 puncta (n = 6 WT; n = 6 AT-1 sTg; n = 5 AT-1^S113R/+^). (**k**) ERGIC morphology in primary-cultured MEFs using ERGIC-53 antibody (scale bar, 3 µm) and quantified using Imaris reconstruction of volume and number of puncta (n = 7 WT; n = 7 AT-1 sTg; n = 7 AT-1^S113R/+^). (**l**) Golgi apparatus morphology in primary-cultured MEFs using GM130 antibody (scale bar, 3 µm) and quantified using Imaris reconstruction of volume and area (n = 11 WT; n = 12 AT-1 sTg; n = 12 AT-1^S113R/+^). (**m**) Representative electron microscopy of MEFs from WT, AT-1 sTg, and AT-1^S113R/+^. Yellow outlines Golgi structures and orange outlines disorganized secretory structures and vesicles. (scale bar, 500 nm). MEFs from multiple cells from 3 biologically independent animals for each genotype for (**b**–**l**). One-way ANOVA. *P < 0.05; **P < 0.005.

Next, we used mouse embryonic fibroblasts (MEFs) from the above mouse models to determine whether identified proteomic changes impact the functional organization of the secretory pathway. Structure-illumination microscopy (SIM) revealed enlarged cisternae within the rough ER of AT-1 sTg MEFs (Fig. 2a). Successfully folded ER transiting proteins are captured at specialized ER structures referred to as ER exit sites (ERES) and then anterogradely transported by COPII-coated transport carriers, which emerge from transitional ER, to the ERGIC and then the Golgi apparatus^20–22^. To determine whether the expansion of the rough ER coincided with increased formation of ERES/COPII structures, we probed for several members of the COPII coat, including Sec13, Sec31, Sec23, Sec24, and Sec16. However, we did not detect major differences in the number of individual puncta (Fig. 2b–f, Supplementary Fig. S1a-d), nor in the co-localization of Sec13 and Sec31 (Fig. 2g, Supplementary Fig. S1a), which reflect the formation of the outer COPII coat^19^, or the co-localization Sec23 and Sec24 (Fig. 2h, Supplementary Fig. S1b), which reflect the formation of the inner COPII coat^23^. Similarly, there were no changes in co-localization of Sec31 and Sec 24 (Fig. 2i, Supplementary Fig. S1c) or Sec13 and Sec16 (Fig. 2j, Supplementary Fig. S1d), which reflect assembly of the inner coat to the outer coat as well as budding events at the transitional ER^24^. Overall, these results suggest that there are no major changes in number nor assembly of COPII cargo structures in either of the AT-1 models.

Next, we used SIM with anti-ERGIC-53 and anti-GM130 antibodies to determine structural changes at the level of the ERGIC and Golgi apparatus, respectively. The results showed a marked expansion of the ERGIC compartment in AT-1 sTg (Fig. 2k) and a significant reduction in volume and surface area of the Golgi apparatus in AT-1^S113R/+^ (Fig. 2l). The above SIM findings were supported by the presence of numerous vesicles in close proximity of the ER and Golgi apparatus resembling ERGIC-structures in AT-1 sTg mice, and disorganized and somewhat smaller Golgi structures in AT-1^S113R/+^ mice as visualized by electron microscopy (Fig. 2m).

Collectively, the above microscopy-based studies revealed expansion of the ER and ERGIC in AT-1 sTg and reduced Golgi apparatus in AT-1^S113R/+^ mice. Overall, they support the conclusion that changes in cytosol-to-ER flux of acetyl-CoA, as determined by increased or reduced AT-1 activity, regulate the organization of the secretory pathway.

### Altered AT-1 activity disrupts normal protein trafficking

To determine whether the above structural reorganization of the secretory pathway highlights changes in protein trafficking within the AT-1 models, we used a previously characterized inducible ER release system that employs tandem repeats of the conditional aggregation domain (CAD), FKBP (F_M_), fused to a protein of interest^25,26^. The F_M_ domain dimerizes in the ER and inhibits transport out of the ER. This dimerization must be solubilized with a CAD ligand for the protein of interest to be released from the ER.

**Figure 3:**
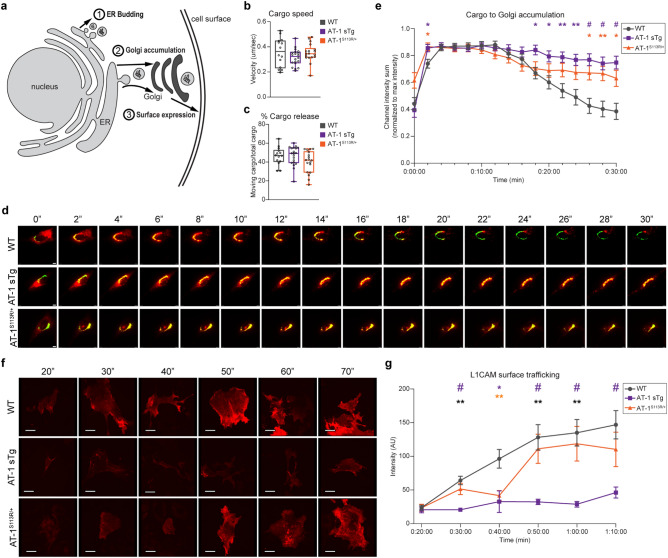
Aberrant AT-1 models demonstrate altered protein trafficking through the secretory pathway. (**a**) Schematic of three experiments used to assess protein trafficking in the secretory pathway. Experiment 1 assesses protein-laden carriers following release from the ER; solubilizer-dependent cargo fluoresces for visualization. Experiment 2 quantifies the time for ER cargo to accumulate in the Golgi apparatus and then egress; solubilizer-dependent DsRed cargo traffics to GFP-labeled Golgi. Experiment 3 quantifies the time for ER cargo to reach the cell surface; solubilizer-dependent cargo with a HaloTag ligand is tracked for 70 min with HaloTag cell impermeable dye added prior to imaging. (**b,c**) Maximum speed of cargo release (**b**), and the percentage of cargo release (**c**), are tracked for 3 min after solubilizer induced cargo release in MEFs (MEFs from multiple cells from 3 biologically independent animals for each genotype n = 14 WT; n = 20 AT-1 sTg; n = 18 AT-1^S113R/+^; MEF cells from 3 biologically independent animals for each group for (**b**,**c**). Student’s T-test. (**d**) Representative cargo (red) accumulation in the Golgi (green) every 2 min, for 30 min in MEFs from WT, AT-1 sTg, and AT-1^S113R/+^ (scale bar, 40 µm). (**e**) Cargo accumulation in the Golgi over 30 min in primary-cultured MEFs (MEFs from multiple cells from 3 biologically independent animals for each genotype at each time point, n = 27 WT; n = 22 AT-1 sTg; n = 23 AT-1^S113R/+^). Mean with SEM are represented at each time point. (**f**) Representative surface trafficking of L1CAM in MEFs from WT, AT-1 sTg, and AT-1^S113R/+^ (scale bar, 40 µm). (**g**) L1CAM intensity at the cell surface in MEFs (MEFs from multiple cells from 3 biologically independent animals for each genotype at each time point, n = 42–76 WT; n = 10–27 AT-1 sTg; n = 19–34 AT-1^S113R/+^). Mean with SEM are represented at each time point. Two-way ANOVA with multiple comparisons at each time point for (**e**,**g**). Purple and orange statistics indicate differences between AT-1 sTg and WT, and between AT-1^S113R/+^ and WT, respectively. Grey statistics indicate difference between AT-1 sTg and AT-1^S113R/+^. *P < 0.05; **P < 0.005; ^#^P < 0.0001.

To examine the initial budding events at the ER (Fig. 3a, Exp. 1), we used 4×F_M_-mCh-NL1^26^, such that neuroligin bound to mCherry could be visualized as it exited the ER. Using this strategy, we did not observe differences in either cargo velocity (Fig. 3b) nor percent of cargo release (Fig. 3c) between WT and either AT-1 model. Consistent with the microscopy of COPII proteins (Fig. 2b–j, Supplementary Fig. S1a-d), these results indicate that the formation of COPII structures from transitional ER is overall preserved in both AT-1 models.

Next, to assess ER-to-Golgi trafficking (Fig. 3a, Exp. 2), we used the DsRedExpress2-FKBP(LV)-GalNAcT2-msGFP2 construct expressing a DsRed cargo protein and a GFP bound GalNAcT, which resides in the *trans*-Golgi. Prior to release, the distribution of DsRed in the ER and the GFP in the Golgi apparatus did not substantially overlap indicating successful sequestration of the two probes. By 2–4 min following CAD availability, all genotypes showed accumulation in the Golgi apparatus. However, at 18 min and beyond, the WT showed cargo dispersion from the Golgi apparatus, whereas the AT-1 sTg and AT-1^S113R/+^ did not (Fig. 3d,e), highlighting a delay in exiting the Golgi apparatus across both AT-1 models.

Finally, to assess protein trafficking to the plasma membrane (Fig. 3a, Exp. 3), we used a 4xF_M_-HaloTag-L1CAM construct containing a HaloTag, which is labeled with a cell impermeable dye at the cell surface. DDS was added to initiate cargo release and cells were visualized every 10 min from 20 to 70 min post-release. In WT MEFs, the intensity of cell surface L1CAM expression steadily climbed with time (Fig. 3f,g), with the steepest slope from 20–50 min, and reaching maximum intensity from 50 to 70 min (Fig. 3f,g). By contrast, in AT-1^S113R/+^ MEFs, L1CAM showed a delay in trafficking to the cell surface up to 40 min and then nearly returned to WT levels at 50–70 min (Fig. 3f,g). Most importantly, AT-1 sTg MEFs remained well below WT across the entire experimental settings (Fig. 3f,g) displaying a significant delay in L1CAM delivery to the cell surface.

The above results indicate that both AT-1 models can efficiently transport nascent glycoproteins from the ER to the Golgi apparatus but experience important alterations when transitioning through the Golgi apparatus. Particularly significant is the delay in Golgi-to-cell surface transport observed in AT-1 sTg mice. These data raise the question of whether defective post-translational processing of N-glycosylated cargo proteins within the Golgi apparatus is responsible for the defective transition and delivery.

### Aberrant AT-1 models display defect in Golgi-dependent N-glycan modification and a shift in the quality of the secretome

The initial GlcNAc_2_Man_9_Glc_3_ oligosaccharide structure that is added within the ER by the OST undergoes major modifications as glycoproteins move through the ER and Golgi apparatus. Specifically, the three terminal glucose are removed and the high-mannose structure is trimmed to allow final modification, which includes addition of fucose and galactose in the *cis/medial*-Golgi and sialic acid in the *trans*-Golgi and *trans*-Golgi network^27^. Importantly, the oligosaccharide chains define much of the functions and activity of glycoproteins^28–30^. Therefore, the complexity of the oligosaccharide chains can serve as a marker of efficient transition through the Golgi apparatus as well as a direct measure of the quality of the secretome (Supplementary Fig. S2).

In order to resolve the quality of the secretome, we developed a new integrated workflow that allows global analysis of the N-glycoproteome in the tissue. The approach uses sequential hydrophilic interaction chromatography (HILIC) for glycopeptide enrichment, coupled to electron-transfer higher-energy collision dissociation (EThcD)^31^. HILIC allows specific and enhanced enrichment of N-glycopeptides prior to LC–MS/MS analysis while EThcD allows highly confident site-specific characterization of intact N-glycopeptides by incorporating fragment ions that result from both glycan and peptide dissociation into one spectrum.

Both human^9–13^ and mouse^4,5^ data indicate that the nervous system is particularly vulnerable to changes in AT-1 activity. Furthermore, neurons respond to the overexpression of AT-1 by altering the expression of their proteome, and building more dendrites and synaptic terminals that heavily rely on the engagement of the secretory pathway^5^. Therefore, we decided to investigate the quality of the N-glycoproteome in the brain of AT-1 sTg and AT-1^S113R/+^ mice.

**Figure 4:**
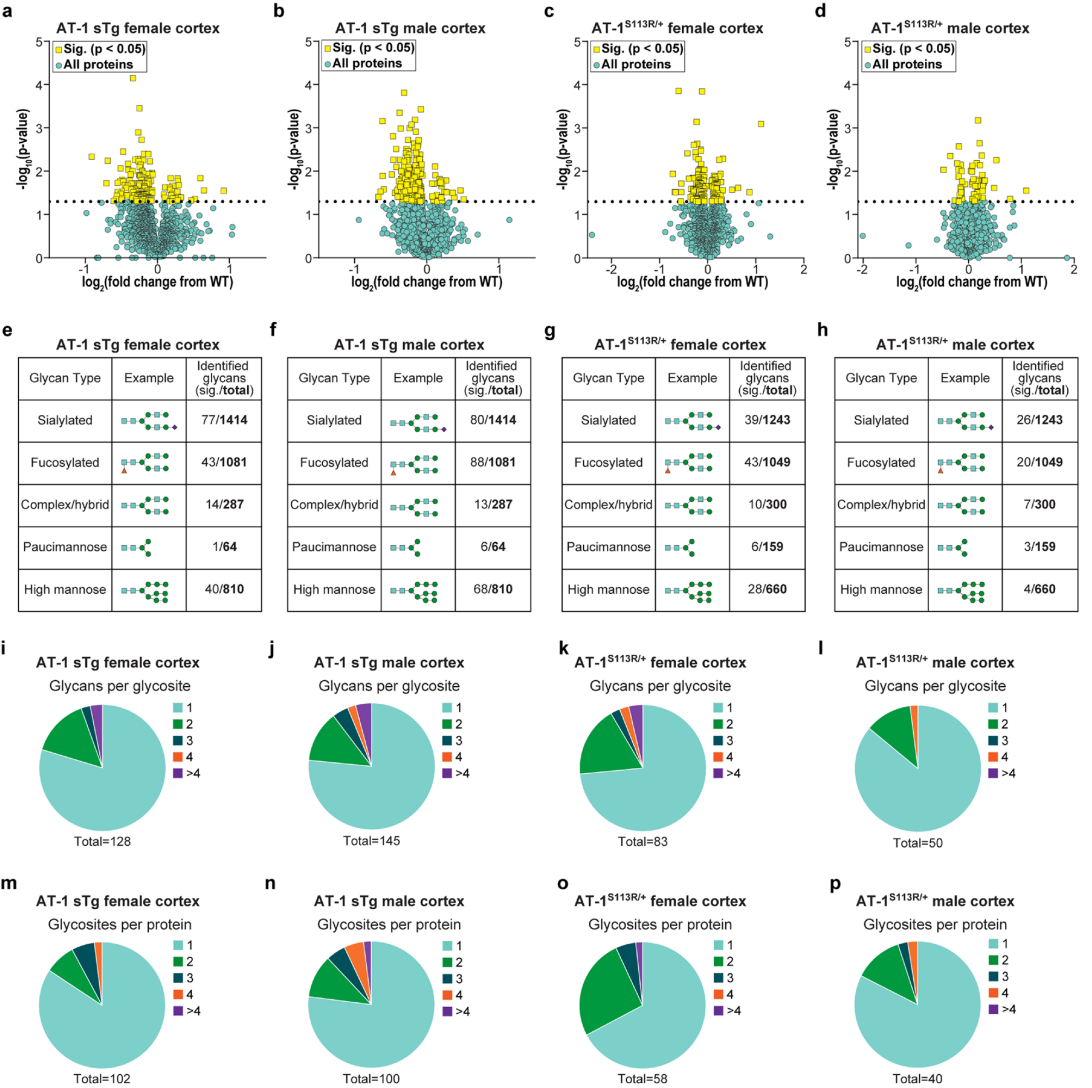
AT-1 sTg and AT-1^S113R/+^ display N-glycoproteomic changes across many cortical glycoforms. (**a**,**b**) Volcano plot displaying all quantified glycoproteins in the cortex of AT-1 sTg (**a**) female (n = 3) and (**b**) male (n = 3), compared with age-matched WT littermates. Statistically significant proteins (175 in female; 255 in male) are highlighted in yellow, and all other proteins are designated in blue. Student’s T-test, P < 0.05. (**c**,**d**) Volcano plot displaying all quantified glycoproteins in the cortex of AT-1^S113R/+^ (**c**) female (n = 3) and (**d**) male (n = 3), compared with age-matched WT littermates. Statistically significant proteins (126 in female; 60 in male) are highlighted in yellow, and all other proteins are designated in blue. Student’s T-test, P < 0.05. (**e**–**h**) Identified glycans are categorized into five glycan types, and are divided by significant over total identified in cortical (**e**) AT-1 sTg female, (**f**) AT-1 sTg male, (**g**) AT-1^S113R/+^ female, and (**h**) AT-1^S113R/+^ male. (**i**–**l)** Significant glycans per glycosite are identified in cortical (**i**) AT-1 sTg female, (**j**) AT-1 sTg male, (**k**) AT-1^S113R/+^ female, and (**l**) AT-1^S113R/+^ male. (**m**–**p**) Significant glycosites per protein are identified in cortical (**m**) AT-1 sTg female, (**n**) AT-1 sTg male, (**o**) AT-1^S113R/+^ female, and (**p**) AT-1^S113R/+^ male.

We found significant changes in 175 and 255 glycoforms of the female (Fig. 4a) and male (Fig. 4b) AT-1 sTg cortex, and in 126 and 60 glycoforms of the female (Fig. 4c) and male (Fig. 4d) AT-1^S113R/+^ cortex. A similar outcome was observed in the hippocampus with 180 and 112 glycoforms affected in female and male AT-1 sTg mice, and 179 and 214 glycoforms affected in female and male AT-1^S113R/+^ mice (Supplementary Fig. S3a-d).

N-glycans were categorized based on their biological maturity, from least mature to most mature: high mannose, paucimannose, complex/hybrid, fucose, and sialic acid (Supplementary Fig. S2). Interestingly, most of the changes in glycosylation profile that we observed in both cortex and hippocampus appeared to occur at the Golgi interface (Fig. 4e–g and Supplementary Fig. S3e-h; see also Supplementary Fig. S2). These alterations could not be attributed to immediate changes of the glycosylation machinery within the Golgi apparatus, as reflected by the mRNA levels of the fucose transporter (SLC35C1), fucosyltransferase (FUT8), sialic acid transporter (SLC35A1), sialyltransferases (ST3GAL3, ST6GAL1, ST6GAL2), galactose transporter (SLC35A2), nor the galactosyltransferases (B4GALT2, B4GALT4) (Supplementary Fig. S4).

In order to evaluate the heterogeneity of the glycoproteome, we quantified significantly changed glycans per glycosite (Fig. 4i–l). The AT-1 sTg male and AT-1^S113R/+^ female showed the highest heterogeneity, with 23% and 27% (respectively) of glycosites having more than one glycan structure change significantly on a glycosite. AT-1^S113R/+^ female also showed the highest proportion of multiple glycosites changing on a single protein (33% of the proteins showed more than one glycosite being affected). However, the heterogeneity of multiple glycosites on one protein being affected was ubiquitous across both models and sexes (Fig. 4m–p). These results were relatively consistent in the hippocampus (Supplementary Fig. S3i-p). Collectively, these findings illustrate the heterogeneous and complex nature of the N-glycan modifications caused by changes in AT-1 activity; multiple glycans on a glycosite are altered, as are the number of glycosites per protein.

**Figure 5:**
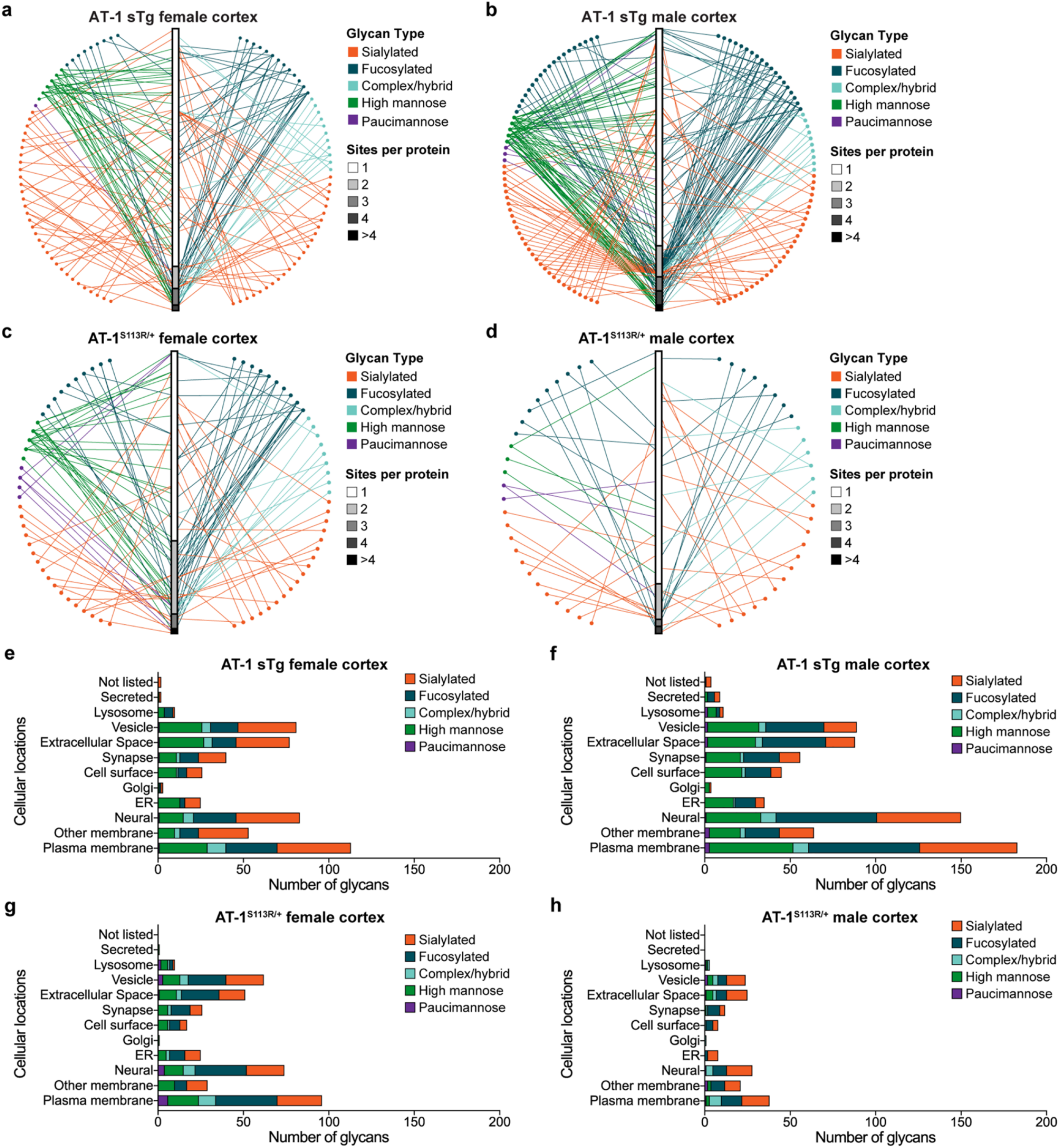
AT-1 sTg and AT-1^S113R/+^ display a highly heterogeneous cortical secretome. (**a**–**d**) Network of significant glycan types plotted to glycoprotein for cortical (**a**) AT-1 sTg female, (**b**) AT-1 sTg male, (**c**) AT-1^S113R/+^ female, and (**d**) AT-1^S113R/+^ male. Circle represents glycan type, with each node representing a specific glycan tree. Glycans intersect with their protein, organized by the number of glycosites identified on that glycoprotein. (**e**–**h**) Glycosylation distribution according to subcellular localizations as defined by GO cellular component terms in cortical (**e**) AT-1 sTg female, (**f**) AT-1 sTg male, (**g**) AT-1^S113R/+^ female, and (**h**) AT-1^S113R/+^ male.

To gain insight into glycosite specific changes, we examined glycan type distribution as they pertained to the number of sites affected per protein. AT-1 sTg female cortex displayed an even distribution of sialic acid, whereas fucose, high mannose, and complex/hybrid species showed a higher appearance on multiple sites per protein (Fig. 5a). These findings might highlight changes in specificity of individual glycosylation events imparted upon by defective transition of the glycoproteins within the Golgi apparatus. Comparing the female cortex of AT-1 sTg to AT-1^S113R/+^ mice, again the majority of fucose and complex/hybrid appear on more than one site per protein; however, sialic acid and high mannose show an even distribution between one site and more than one site per protein (Fig. 5c). AT-1 sTg male cortex shows that the majority of fucose and complex/hybrid glycans are on more than one glycosite per protein, whereas high mannose and sialic acid display an even distribution across number of sites (Fig. 5b). The very few glycan types in AT-1^S113R/+^ male cortex show an even distribution of all glycan types except high mannose, which only exists on one site per protein (Fig. 5d). Overall similar results, with only minor differences, were observed in the hippocampus across models (Supplementary Fig. S5).

To understand the biological relevance of the changes observed within the entire secretome, all glycoforms were categorized into subcellular locations based on their GO Cellular Compartment terms. Across both AT-1 models and sexes, the subcellular locations which showed the greatest changes include vesicle, extracellular space, neural, and plasma membrane (Fig. 5e–h). Similar results were observed in the hippocampus (Supplementary Fig S5e,f,g,h). Albeit with some intrinsic differences, most of the significant changes observed across genetic models, brain regions, and sexes were accounted for by fucose, sialic acid and high mannose (Fig. 5e–h and Supplementary Fig S5e,f,g,h). Furthermore, across many of these analyses, we found a substantial amount of high mannose structures among proteins that are clustered within the plasma membrane and cell surface subgroups. Therefore, Golgi-dependent glycosylation events appear to be more affected and cellular compartments/pathways that depend on successful Golgi-to-plasma membrane transport appear to be more dramatically affected within our genetic models.

**Figure 6:**
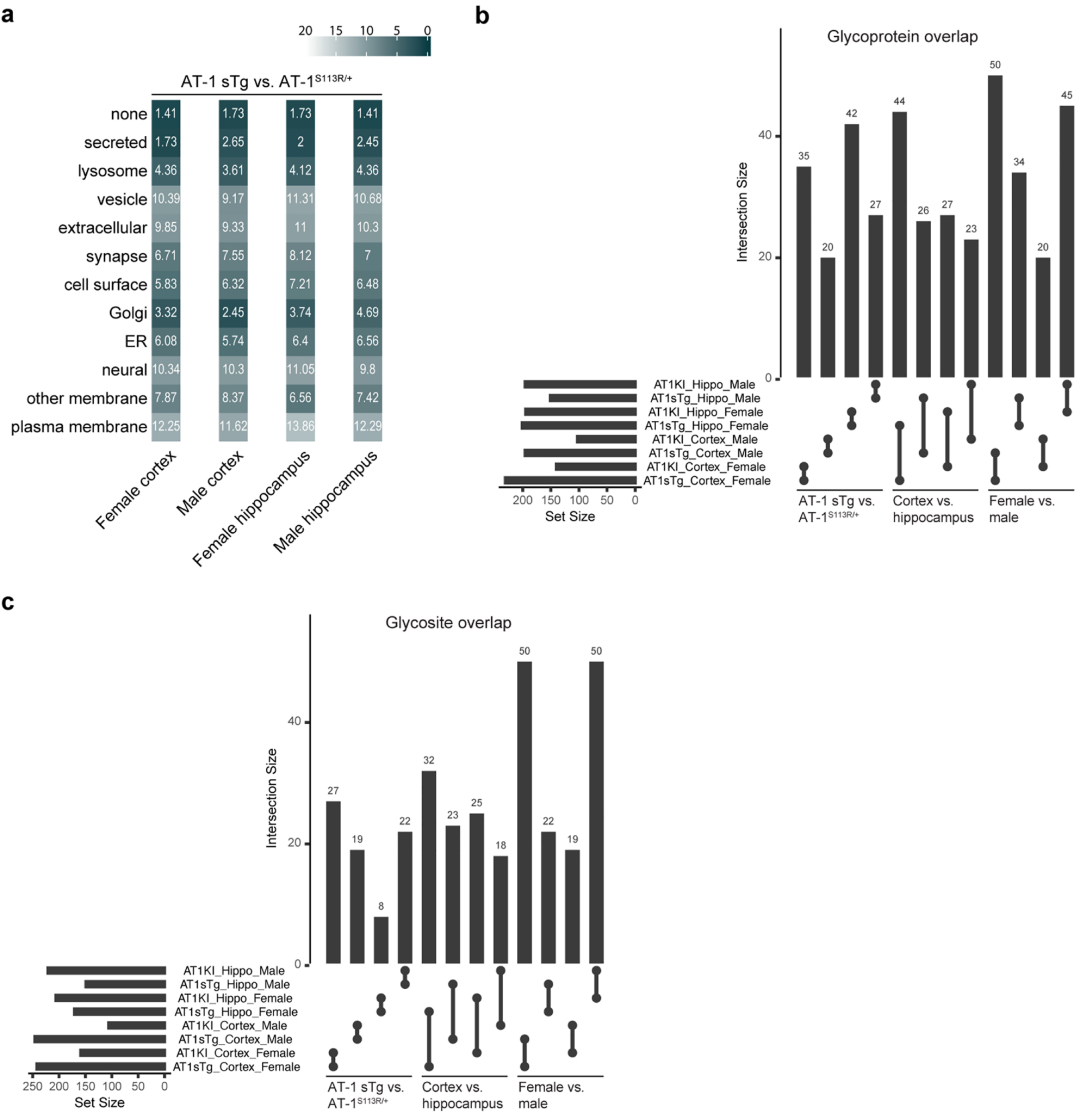
AT-1 sTg and AT-1^S113R/+^ display both divergent and convergent N-glycoproteomic changes across subcellular locations. (**a**) Euclidean distances comparing the distance between significant glycoforms found in AT-1 sTg and AT-1^S113R/+^ , broken down by sex and brain region. Distances were calculated according to their subcellular locations, with higher similarity indicated with dark colors. (**b**,**c**) Significant glycoprotein (**b**) and glycosite (**c**) overlap is examined at the level of model (AT-1 sTg v. AT-1^S113R/+^), brain region (cortex vs. hippocampus) and sex (female vs. male). The upper bar graph indicates the size of intersection and the left bar graphs shows total number of significant glycoproteins (**b**) or glycosites (**c**) in each dataset.

The adaptations addressed thus far indicate that similar pathways and subcellular locations are being affected in AT-1 sTg and AT-1^S113R/+^. To determine whether there was concordance across all groups, we examined overlapping glycoforms according to their subcellular location, considering whether the glycoprotein had an increase or decrease expression when compared with WT. Interestingly, the most divergent (opposite) changes between AT-1 sTg and AT-1^S113R/+^ included vesicle, extracellular, neural, and plasma membrane compartments, which, as highlighted before, depend on successful Golgi-to-plasma membrane transport (Fig. 6a). Conversely, the most convergent changes were found in the lysosome and Golgi subgroups (Fig. 6a). These differences and similarities were conserved across sex and brain region thus highlighting a specific biological response.

When analyzed for glycoprotein and glycosite, we observed 28% (female) and 17% (male) convergent changes in glycoproteins and 14% (female) and 10% (male) convergent changes in glycosites across the cortex of AT-1 sTg and AT-1^S113R/+^ mice (Fig. 6b,c). A similar trend was observed in the hippocampus with a 35% (female) and 20% (male) overlap in altered glycoproteins, but only a 4% (female) and 12% (male) overlap in the glycosites affected (Fig. 6b,c). When we compared overlap between cortex with hippocampus, AT-1 sTg mice showed a 32% (female) and 19% (male) overlap in glycoproteins, and a 15% (female) and 11% (male) overlap in glycosites (Fig. 6b,c). Similarly, AT-1^S113R/+^ mice showed a 24% (female) and 19% (male) overlap in glycoproteins, and a 13% (female) and 11% (male) overlap in glycosites.

### Aberrant AT-1 models display expansion of the lysosomal network

Under normal conditions, a terminal mannose-6-phosphate (M6P) unit serves as a recognition signal for lysosomal-targeted enzymes. Sorting is mediated by two specific M6P receptors (M6PRs), which bind to M6P in the Golgi apparatus and deliver lysosomal enzymes to the lysosomes^32,33^. The drastic expansion of high-mannose structures lacking a terminal glucose observed in AT-1 sTg mice can potentially disrupt normal M6PR-mediated functions resulting in mistargeting of non-lysosomal cargo glycoproteins from the Golgi apparatus to the lysosomes thus overloading the lysosomal network.

**Figure 7:**
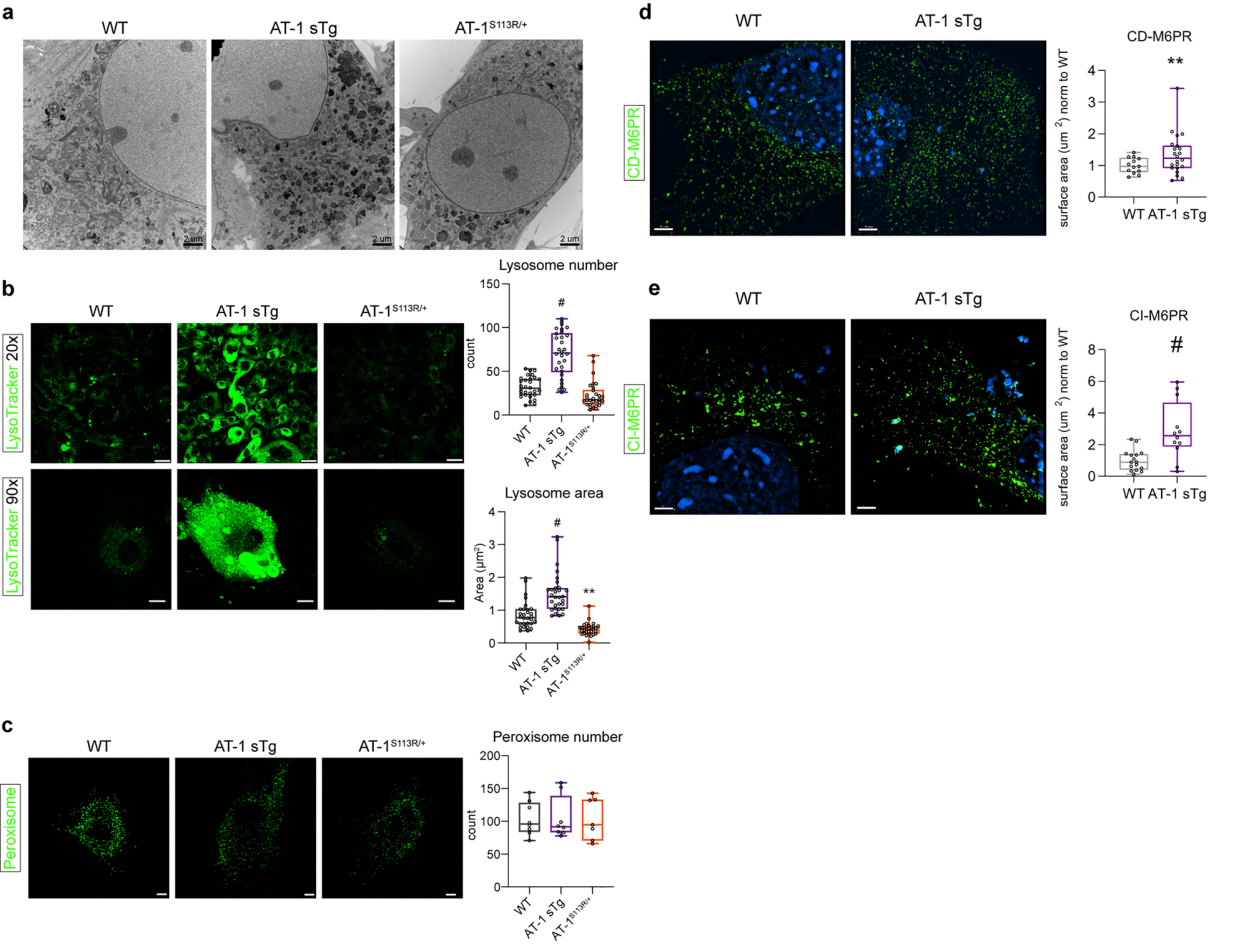
AT-1 sTg and AT-1^S113R/+^ display alterations in the CD-M6PR, CI-M6PR, and lysosomal networks. (**a**) Representative electron microscopy of MEFs from WT, AT-1 sTg, and AT-1^S113R/+^ (scale bar, 2 µm). (**b**) Lysosomal morphology in primary-cultured MEFs using LysoTracker stain (20 × scale bar, 30 µm; 90 × scale bar, 15 µm) and quantified using Imaris reconstruction of surface area and number of puncta (n = 30 WT; n = 30 AT-1 sTg; n = 30 AT-1^S113R/+^). (**c**) Peroxisome morphology in primary-cultured MEFs using Peroxisome CellLight stain (scale bar, 10 µm) and quantified using Imaris reconstruction of number of puncta (n = 8 WT; n = 8 AT-1 sTg; n = 7 AT-1^S113R/+^). (**d**) Representative CD-M6PR in primary cultured MEFs using CD-M6PR antibody (scale bar, 3 µm). Surface area was quantified using Imaris reconstruction (n = 13 WT; n = 22 AT-1 sTg). (**e**) Representative CI-M6PR in primary cultured MEFs using CI-M6PR antibody (scale bar, 3 µm). Surface area was quantified using Imaris reconstruction (n = 16 WT; n = 12 AT-1 sTg). Two-way ANOVA (**b**,**c**). Welchs’ t test (**d**,**e**). **P < 0.005; ^#^P < 0.0001.

To determine whether this was indeed the case, we used EM and studied MEFs from AT-1 sTg and AT-1^S113R/+^ mice. Consistent with the above hypothesis, we observed a striking expansion of the lysosomal network in the AT-1 sTg model, where the cytosol appeared loaded with electron dense organelles (Fig. 7a). To confirm the identity of these organelles, we used the lysosomal-specific probe, LysoTracker. We observed a marked increase in both number and area of lysosomes in AT-1 sTg MEFs when compared to WT MEFs, thus highlighting the expansion of the lysosomal network (Fig. 7b). Conversely, AT-1^S113R/+^ MEFs displayed significantly smaller lysosomes than WT (Fig. 7b). No differences across genotypes were observed when we labeled the cells with a peroxisomal probe, used as negative control.

Finally, assessment of M6PR in AT-1 sTg MEFs revealed a significant expansion of both the cation-dependent (CD) and cation-independent (CI) networks suggesting increased cycling of CD-M6PR and CI-M6PR between the Golgi apparatus and the endosomal/lysosomal compartments (Fig. 7d,e).

## Discussion

Both cell- and mouse-based studies indicate that the ER acetylation machinery is essential to maintain proteostasis within the ER. Essential features include: (1) a QC-like process where acetylation of correctly folded glycoproteins allows them to successfully leave the ER and engage the secretory pathway; and (2) disposal of toxic protein aggregates through the induction of reticulophagy. The former process requires ATase1 and ATase2 to interact with the OST while the nascent polypeptide enters the organelle; the latter requires tight regulation of the acetylation status of ATG9A, which is essential for the engagement of FAM134B and SEC62 and the activation of the LC3β^6,34,35^.

Acetyl-CoA, which serves as the donor for acetylation in the ER, is synthesized in the cytosol and must be transported across the ER membrane by AT-1. In this study, we used AT-1 sTg and AT-1^S113R/+^ mice with increased and reduced AT-1 activity, respectively, to study how the secretory pathway adapts to changes in cytosol-to-ER flux of acetyl-CoA. Through the analysis of the proteome and acetyl-proteome, we determined that many proteins involved with essential ER features, such as protein biosynthesis and insertion within the ER, post-translational modification, folding, QC and ER-to-Golgi trafficking were altered. These changes were paralleled by significant reorganization of the secretory pathway. Specifically, AT-1 sTg mice displayed reorganization of the ER and expansion of the ERGIC network, while AT-1^S113R/+^ mice displayed a contraction of the Golgi apparatus. Furthermore, these morphological changes were paralleled by evident changes in the trafficking of newly-synthesized glycoproteins out of the ER. Particularly significant was the delay in Golgi-to-plasma membrane transport observed in AT-1 sTg mice. Analysis of the N-glycoproteome revealed significant and widespread changes in both AT-1 sTg and AT-1^S113R/+^ mice. Importantly, proteins that showed the most significant and divergent changes across genetic models were assigned to trafficking vesicles, extracellular space, neural, and plasma membrane compartments, all requiring post-translational maturation within the Golgi apparatus and successful post-Golgi transport across the secretory pathway. Furthermore, the great majority of changes involved Golgi-specific glycan species thus highlighting a possible defect in the ability of the nascent glycoprotein to successfully transition in a progressive fashion through the N-glycan modification machinery of the Golgi apparatus. The apparent defects in Golgi-specific N-glycan modification as well as delayed Golgi-to-plasma membrane transition observed in AT-1 sTg mice was accompanied by a striking expansion of the M6PR and lysosomal networks, which likely involves mistargeting of glycoproteins with high-mannose structures lacking a terminal glucose by the M6PR.

Overexpression of AT-1 in cellular systems is accompanied by increased incorporation of O-propargyl-puromycin and azide-modified mannosamine, highlighting both increased protein biosynthesis and increased terminal sialylation^5,6^. Furthermore, neuronal overexpression of AT-1 caused a significant expansion of the proteome with 476 proteins found to be upregulated; an effect that was connected to the expansion of the dendritic and synaptic network^5^. The results described here suggest that in AT-1 sTg the expansion of the ER and the ERGIC, together with the increase in ER-to-Golgi transport of cargo material is not paralleled by compensatory changes within the Golgi apparatus to ensure post-translational N-glycosylation. It is also possible that the increased availability of acetyl-CoA within the ER, as caused by overexpression of AT-1 might affect the activity and specificity of the ATases thus resulting in incorrectly folded glycoproteins to be acetylated and forced to transition toward the Golgi apparatus. Indeed, individual acetyltransferases possess different *K*m values within the normal physiological range of acetyl-CoA, and changes or fluctuations in the concentrations of acetyl-CoA can reduce or promote the acetylation of specific lysine residues^1,36^. Furthermore, the analysis of the proteome identified significant changes within the chaperone sub-cluster, perhaps reflecting increased abundance of misfolded glycoproteins in the ER. If indeed the changes in post-translational maturation of N-glycoproteins reflect the aberrant transition of incorrectly folded glycoproteins to the Golgi apparatus, we could argue that the delay in Golgi-to-plasma membrane transition and increased diversion of cargo material to the lysosomal compartment is part of a QC-like event within the Golgi apparatus attempting to prevent successful transport of incorrectly folded polypeptides to their final destination. This would explain why we observed minimal changes in the glycosylation status of Golgi resident proteins. Interestingly, down-regulation of the Golgi-based CMP-sialic acid and GDP-fucose transporters impedes successful Golgi-based glycosylation and prevents Golgi-to-cell surface transport of glycoconjugates^37^. This block in protein trafficking is paralled by induction of ER stress and inhibition of protein translation, suggesting that active crosstalk between the ER and the Golgi apparatus regulates dynamics of the secretory pathway^37^.

Overall, these results support the conclusion that the cytosol-to-ER flux of acetyl-CoA can affect organelle dynamics across the secretory pathway with widespread consequences on Golgi-specific post-translational glycosylation and the quality of the secretome. Therefore, acetyl-CoA homeostasis, impinged upon by the ER acetylation machinery, has far reaching consequences in metabolic reprogramming across cellular compartments.

## Methods

### Animals

All animal experiments were carried out in accordance with the NIH Guide for the Care and Use of Laboratory Animals, and received ethical approval by the Institutional Animal Care and Use Committee of the University of Wisconsin-Madison. AT-1^S113R/+^ animals were generated by crossing mice carrying the Slc33a1-S113R mutation to WT animals^4^. AT-1^S113R/+^ animals were studied between 2.5 to 3.5 months of age. Generation of AT-1 sTg mouse was achieved by crossing Rosa26:tTA mice with pTRE AT-1 mice to generate ROSA26:tTA;pTRE- AT-1 (AT-1 sTg mice)^6^. AT-1 sTg mice were studied at 2.5 to 3.5 months of age. Age-matched wild-type (WT) littermates were used as controls. Males were used for the proteome and acetyl-proteome, as described previously^14^. Males and females were used for all other experiments in this study.

### Mouse embryonic fibroblast isolation

Isolation of mouse embryonic fibroblasts, MEFs, were described previously^4^. Briefly, embryos were collected from pregnant females at embryonic day 12.5 to 13.5. Embryos (without heads or visceral organs) were minced in sterile EDTA (0.25%; Mediatech), then incubated in a 37 °C CO_2_ incubator for 30–45 min. Complete MEF media (DMEM-high glucose, 10% FBS, penicillin/streptomycin/glutamine mixture, fungizone) was used to quench the trypsin; tissue was further broken by gentle pipetting. Cells were spun at (1000 rpm for 5 min), and the supernatant was discarded. Cells were washed again in MEF media, then plated. Confluent cells were passaged at 1:4 dilutions by using trypsin–EDTA.

### Stoichiometry of acetylation

Quantification of acetylation stoichiometry follows methods described previously, with the following modifications^14,38,39^. Liver protein samples (200 μg) from mitochondrial and cytosolic subcellular fractions were denatured in urea buffer (8 M urea (deionized), 100 mM ammonium bicarbonate (pH = 8.0), 5 mM DTT) and incubated at 60 °C for 20 min^40^. Cysteines were alkylated with 50 mM iodoacetamide, then incubated for 20 min. Two rounds of ~ 20 µmol heavy isotopic D_6_-acetic anhydride (Cambridge Isotope Laboratories) chemically acetylated the samples, which were then diluted to 2 M urea using 100 mM ammonium bicarbonate (pH = 8.0) before digestion with 1:100 trypsin at 37 °C for 4 h. Before a second digestion with 1:100 gluC, samples were diluted to 1 M urea. Chemically acetylated peptides were fractionated into 6 fractions using a Shimadzu LC-20AT HPLC system with a Phenomenex Gemini NX-C18 column (5 µm, 110Å, 150 × 2.0 mm). Data-independent acquisition (DIA) analysis was conducted by a Thermo Q-Exactive Orbitrap coupled to a Dionex Ultimate 3000 RSLC nano UPLC with a Waters Atlantic reversed phase column (100 μm × 150 mm). A spectral library containing all light and heavy acetyl-lysine feature pairs was generated to deconvolute and analyze the DIA spectra using the openly available MaxQuant (v1.6.1) software package. Spectral library samples were treated with C^12^-acetic anhydride (Sigma), but were otherwise processed identically to the experimental samples and analyzed using data dependent acquisition (DDA) mass spectrometry analysis. A combined library was formed from DDA runs of both the mitochondrial and cytosolic fractions. Heavy acetyl fragment ion pairs were generated in silico, such that the spectral library would contain both the light (endogenous) acetylation peaks and the heavy (chemical) acetylation peaks. Spectronaut (v10) was used to process the experimental samples using the generated spectral library. The subcellular fraction experimental samples were processed separately using an in-house R script, which can be accessed through the GitHub link: [http://doi.org/10.5281/zenodo.3238525]; stoichiometry was calculated using the ratio of endogenous (light) fragment ion peak area over the total (endogenous and chemical) fragment ion peak area. All fractions were combined for downstream analysis. Proteins were filtered as significant changes if *P* < 0.05 compared to WT. The raw data, processed data, spectral library, and the analysis logs detailing the Spectronaut analyses settings have been deposited to the ProteomeXchange Consortium via the MassIVE partner repository with the dataset identifier PXD014013 [http://proteomecentral.proteomexchange.org/cgi/GetDataset?ID=PXD014013].

### Quantitative proteomics

Quantification of the proteome was described previously^14^. Briefly, liver samples of cytosol, mitochondria, and nucleus were homogenized, then lysed (8 M urea, 50 mM Tris, pH = 8, 5 mM CaCl_2_, 20 mM NaCl, 1 EDTA-free Roche protease inhibitor tablet and 1 Roche PhosSTOP phosphatase inhibitor tablet) with a probe sonicator. Crude lysates were centrifuged (14,000×*g*; 5 min), and supernatant protein concentrations were measured by Pierce BCA Protein Assay (Pierce, Rockford, IL). 400 μg of protein lysate was reduced in 5 mM dithiothreitol (DTT), followed by alkylation in 15 mM iodoacetamide (IAA), quenched by adding DTT to 5 mM, and diluted with Tris buffer (pH = 8) to 0.9 M urea. Proteins were digested with trypsin (Promega, Madison, WI), and quenched by adding trifluoroacetic acid (TFA) to a final concentration of 0.3% and desalted with C18 SepPak cartridges (Waters, Milford, MA). Peptides were vacuum dried and reconstituted in 0.5 M TEAB prior to labeling. Samples were assigned to two batches of 4-plex dimethylated leucine (DiLeu) tags each in biological duplicate. 4 mg of each DiLeu tags were suspended in anhydrous DMF, 4-(4,6-dimethoxy-1,3,5-triazin-2-yl)-4-methyl-morpholinium tetrafluoroborate (DMTMM) and N-methylmorpholine (NMM) at 0.6 × molar ratios, then vortexed and centrifuged. The supernatant was mixed with 400 μg tryptic peptides for each condition. Peptides were labeled at a 10:1 label to peptide mass ratio, then vortexed and quenched by adding 5% NH_2_OH to the final concentration of 0.25%. 4-plex mixtures were purified by strong cation exchange liquid chromatography (SCX LC) with a PolySULFOETHYL A column (200 mm × 2.1 mm, 5 μm, 300 Å, PolyLC, Columbia, MD). Labeled peptides were collected cleaned, and fractionated with a Kinetex C18 column (5 μm, 100 Å, Phenomenex, Torrance, CA), and a binary mobile phase at pH = 10. Peptides were reconstituted in 0.1% formic acid (FA) and subjected to reversed phase LC–MS/MS analysis with an Orbitrap Fusion Lumos Tribrid mass spectrometer (Thermo Fisher Scientific, San Jose, CA) interfaced with a Dionex Ultimate 3000 UPLC system (Thermo Fisher Scientific, San Jose, CA). Peptides were loaded onto a microcapillary column custom-packed with Bridged Ethylene Hybrid C18 particles (1.7 μm, 130 Å, Waters). Labeled peptide were separated with a 90 min gradient. Survey scans of peptide precursors from *m/z* 350 to 1500 were performed and an AGC target of 2 × 10^5^ with a maximum injection time of 100 ms. The top 20 intense precursor ions were selected for HCD fragmentation. Raw files were processed with Proteome Discoverer 2.1 engine (Thermo Fisher Scientific, San Jose, CA) with Byonic search engine (Protein Metrics Inc, San Carlos, CA). Spectra were searched using the Uniprot *Mus musculus* database. DiLeu labels on peptide N-termini and lysine residues (+ 145.12801 Da) and carbamidomethylation on cysteine residues (+ 57.02146 Da) were considered fixed modifications. Identifications were filtered to 1% peptide and protein FDR. Proteome Discoverer was used for quantitation; only the PSMs that contained all reporter ion channels were considered. Reporter ion ratio values for protein groups were exported to Microsoft Excel and all fractions were combined for downstream analysis (see statistics section for processing). Significant proteins were determined using Fisher’s method (*P* < 0.05). The mass spectrometry proteomics data have been deposited to the ProteomeXchange Consortium via the PRIDE partner repository with the dataset identifier PXD013736 [http://proteomecentral.proteomexchange.org/cgi/GetDataset?ID=PXD013736].

### DiLeu labeling and glycoproteomics

12-plex DiLeu^41^ labeling was conducted as previously reported^14^. Briefly, dissected brain region samples of AT-1 sTg, AT-1^S113R/+^ and WT mouse models were homogenized, then lysed in 8 M urea buffer with a probe sonicator. Lysate containing proteins was reduced in 5 mM dithiothreitol (DTT) at room temperature for 1 h, followed by alkylation in 15 mM iodoacetamide (IAA) for 30 min in the dark. Alkylation was quenched by adding DTT to 5 mM. The alkylated protein was diluted and then digested with trypsin (Promega, Madison, WI) at 1:50 enzyme to protein ratio at 37 °C for 18 h. Tryptic peptides were desalted with C18 SepPak cartridges (Waters, Milford, MA), dried under vacuum, and reconstituted in 0.5 M TEAB before labeling.

DiLeu tags were suspended in anhydrous DMF and combined with 4-(4,6-dimethoxy-1,3,5-triazin-2-yl)-4-methyl-morpholinium tetrafluoroborate (DMTMM) and N-methylmorpholine (NMM) at 0.6 × molar ratios to tags. The mixture was vortexed at room temperature for 1 h. Following centrifugation, the supernatant was immediately mixed with tryptic peptides from one condition (10:1 tag to peptide w/w), and vortexed at room temperature for 2 h. The reaction was quenched by NH_2_OH. Each batch of labeled peptides was combined respectively as 12-plex mixtures.

### Glycopeptide enrichment

DiLeu labeled glycopeptides were enriched using in-house packed SAX-HILIC SPE tips following previously reported procedure with minor modification^42,43^. 3 mg of cotton wool was inserted into a 200 µL TopTip. SAX LP bulk material was dispersed in 1% TFA as a 10 mg/200 µL slurry and activated for 15 min under vigorous vortexing. After activation, 60 µL slurry was added to the spin-tip. Solvent was removed by centrifugation at 1200 rpm for 2 min, after which the SAX material was packed at the top of the tip. The stationary phase was then conditioned by 300 µL 1% TFA and 300 µL loading buffer (80% ACN, 1% TFA), each repeated 3 times. 2 mg of DiLeu labeled peptides were aliquoted to 200 µg. Each aliquot was dissolved in 300 µL loading buffer and loaded onto the tips by centrifugation at 1200 rpm for 2 min; the flow through was collected and loaded again to ensure complete retention. The tips were then washed with 300 µL loading buffer for a total of 6 times, after which the four eluted fractions of 300 µL 70% ACN with 0.2% FA, 53% ACN with 0.2% FA, 30% ACN with 0.2% FA and 5% ACN with 0.2% FA were collected in four separate PE tubes. Corresponding fractions from the ten aliquots were pooled and dried under vacuum before MS analysis.

### LC–MS/MS analysis of intact glycopeptides

Enriched glycopeptides in each fraction were reconstituted in 0.1% FA and subjected to reversed phase LC–MS/MS analysis with an Orbitrap Fusion Lumos mass spectrometer (Thermo Fisher Scientific, San Jose, CA) interfaced with a Dionex Ultimate 3000 UPLC system (Thermo Fisher Scientific, San Jose, CA). Peptides were separated on a 15 cm length, 75 μm i.d. custom-packed BEH C18 (1.7 μm, 130 Å, Waters) capillary column with an 80 min gradient from 0 to 30% ACN (0.1% FA). Mass spectrometer was operated in a top 20 data-dependent acquisition mode with HCD-product dependent-EThcD fragmentation^44^. Survey scans of peptide precursors from *m/z* 400 to 2000 were performed at resolving power of 120 K and AGC target of 4 × 10^5^ with a maximum injection time of 150 ms. Tandem MS acquisition was at resolving power of 60 K, AGC target of 5 × 10^4^ and dynamic exclusion of 12 s of 10 ppm mass tolerance. The top 20 intense precursor ions were selected and subjected to HCD fragmentation at a normalized collision energy of 33%. If signature oxomium ions (HexNAc 204.087 m/z, HexNAcHex 366.140 m/z, HexNAc fragments 138.055 m/z and 168.065 m/z) of intact glycopeptides were detected by HCD survey scan, an EThcD hybrid fragmentation was triggered. ETD reaction time was set to 30, 20 or 10 ms when precursor charge states were z = 2, 3–5 or 6–7. HCD supplemental activation energy was 33%. Maximum ion injection times for HCD survey scan and EThcD scan are 125 and 250 ms.

### Glycoproteome data processing

Raw files were processed with Byonic search engine (Protein Metrics Inc, San Carlos, CA) embedded within Proteome Discoverer 2.1 (Thermo Fisher Scientific, San Jose, CA). Spectra were searched against the SwissProt *Mus musculus* proteome database (August 13, 2016; 24,903 entries). Trypsin digestion missed cleavage was set < 3. The parent mass error tolerance was 10 ppm, and fragment mass tolerance was 0.01 Da. Fixed modifications were specified as carbamidomethylation (+ 57.02146 Da) on C residues and 12-plex DiLeu (+ 145.12801 Da) on peptide N-terminus and K. Dynamic modifications included oxidation of M (+ 15.99492 Da, rare1), deamidation (+ 0.984016 Da, rare1) of N or Q, and N-glycosylation (common1). Glycan modifications were specified as Byonic embedded mammalian N-glycan database (309 entries). Identifications were filtered to 1% protein FDR. Gene ontology annotation of glycoprotein and Student’s t-test of glycopeptide quantitation results were performed using Perseus software^45^. Riley et al. reported useful tools to analyze large-scale site-specific glycoproteomics data^46^. Results were further processed by in-house written R scripts. Glycopeptides were exclusively categorized into five glycan type categories based on glycan composition: (1) sialic acid (containing NeuAc/NeuGc), (2) fucose (containing Fucose), (3) complex/hybrid (> 2 NeuAc), (4) high-mannose (2 NeuAc and > 5 Hex), and (5) paucimannose (2 NeuAc and < 5 Hex). The N-glycoproteomics data have been deposited to the Proteome Xchange Consortium via the PRIDE partner repository with the data set identifier PXD019770 [http://proteomecentral.proteomexchange.org/cgi/GetDataset?ID=PXD019770].

### Quantitative post-acquisition data set analyses

The proteome and the acetyl-proteome were analyzed as described previously^14^. Cluster analysis was determined using KEGG pathways which arose during pathway analysis^47,48^. For the secretory pathway-related cluster, all proteins that were found in the following KEGG pathways in either of the AT-1 models and in either of the proteome or acetyl stoichiometry were included: protein processing in the ER, ribosome, proteasome, spliceosome, and RNA transport. These proteins were the input for a STRING analysis. Proteins with no interactions were hidden, with the minimum required interaction score set at high confidence (0.7). Active interaction sources included all sources except textmining.

To determine subcellular localization for glycoproteome data, proteins were categorized according to their Uniprot GO Cellular Component annotation. Proteins were placed inclusively, into the following categories as done previously^14^: “plasma membrane”; “other membrane” (includes GO terms with the word ‘membrane’ exclusive of plasma membrane); “neural” (includes GO terms that contain ‘axon’, ‘neuro’, or ‘myelin’); “ER” (includes any term that contains ‘endoplasmic’); “Golgi” (includes terms that contain ‘Golgi’ and do not contain ‘endoplasmic’); “cell surface” (includes any GO term that includes ‘surface’); “synapse”, “extracellular”, “vesicle”, “lysosome”, and “secreted” (include terms with their respective names); and “none listed” (includes any GO term that did not contain any of the other 11 subcellular groups used). The protein-glycan networks of significantly altered glycopeptides (p < 0.05) were created in R 3.6.0 using the igraph and ggnetwork library. Subcellular locations of glycopeptides were grouped from GO cellular component terms according to the same rules as in (Riley)^46^. Pairwise Euclidean distances between subcellular groups of significant glycopeptides from different quantitative experiments was calculated. Each GO group was considered as a 1707-dimension vector with each glycopeptide as a direction. The dimension vector was constructed by the union set of significantly altered glycopeptides across samples. The magnitude of the dimension is + 1 or − 1 for corresponding up- or down- regulated glycopeptides. Upset plots detailing the overlap between significantly altered glycoproteins and glycosites between datasets were created using the UpSetR package in R Studio. KEGG pathway enrichment and visualization of significant glycoproteins against *Mus Musculus* gene were performed with clusterProfiler library, Benjamini–Hochberg adjustment was performed to an FDR of 0.05^46^.

### Immunocytochemistry

Cultured primary MEF cells were transfected with a plasmid carrying ER3-mCherry fusion protein, KDEL, and ER signal peptide (Gift from Michael Davidson; Addgene Plasmid #55,041) or transfected with CellLight Golgi-GFP BacMam 2.0 (C10592, ThermoFisher Scientific) overnight. The coverslips were then fixed with paraformaldehyde for 10 min, (4%, 15,710, Electron Microscopy Sciences), and permeabilized with 0.1% Triton TM-X100 (Roche Applied Science) for 5 min, followed by an incubation in blocking buffer (10% BSA, 5% goat serum in PBS) for one hour. All antibodies were diluted in antibody dilution buffer (1% BSA, 5% goat serum in PBS) and stained with ERGIC-53 (E1031, Sigma Aldrich, 1:80), Sec13 (A303-980A, Bethyl Laboratories or H00006396-B02P, Novus Biologicals, 1:50), Sec16 (A300-648A, Bethyl Laboratories. 1:50), Sec31A (sc-136233, SantaCruz Biotechnology, 1:50), Sec24C (A304-760A, Bethyl Laboratories, 1:50), Sec23 (ab99552, Abcam, 1:50), GM130 (610,823, BD Bioscience, 1:100), CI-M6PR (ab124767, Abcam, 1:200), or CD-M6PR (ab134153, Abcam, 1:50) for overnight incubation. Cells were washed three times in PBS and incubated with secondary antibodies AlexaFluor488 (A-11034, 1:1000) and AlexaFluor647 (A-21463 or A-21447, 1:1000) for one hour. Cells were washed three times in PBS; Fluoro-Gel II with DAPI (17985-50, Electron Microscopy Sciences) was used to mount the cover slips. Images were acquired using Structured Illumination Microscopy (Nikon SIM), and analyzed in Imaris imaging software (Bitplane, Oxford Instruments) using the Surfaces and Spots modules. Images of cells stained for CI-M6PR and CD-M6PR were acquired using both SIM and confocal microscopy (Nikon A1), and were analyzed using the surfaces module in Imaris imaging software. To stain for peroxisomes, CellLight Peroxisome-GFP, BacMam 2.0 (C10604, ThermoFisher) was used, and to stain for lysosomes, LysoTracker Green DND-26 (L7526, ThermoFisher) was used. Images were acquired using A1R-HD confocal microscope (Nikon) using a 20 × and 60 × water objective.

### Electron microscopy

MEFs were grown on coverslips and fixed in 2.5% glutaraldehyde, 2.0% paraformaldehyde buffered in 0.1 M sodium phosphate buffer (PB) for 1 h at room temperature (RT). The samples were then rinsed 5 × 5 min in PB, and post-fixed in 1% osmium tetroxide and 1% potassium ferrocyanide in 0.1 M PB for 1 h at room temperature (RT), then rinsed in PB as before. Dehydration was performed in a graded ethanol series (35, 50, 70, 80, 90% for 5 min each step, 95% for 7 min, 100% for 3 × 7 min) at RT, and then transitioned in dry acetone 2 × 7 min at RT. Fully dehydrated samples were infiltrated in increasing concentrations of Durcupan ACM (Sigma-Aldrich) and acetone mixtures in the following order: 20 min incubation in 25% PolyBed 812, 75% acetone, 20 min incubation in 50% PolyBed812, 50% acetone, 1 h incubation in 75% PolyBed 812, 25% acetone, and finally a 45 min incubation at 60 °C in 100% PolyBed 812. Embedding and polymerization took place in fresh Durcupan ACM for 24 h at 60 °C. The coverslips were removed with hydrofluoric acid and the samples were sectioned on a Leica EM UC6 ultramicrotome at 100 nm. The sections were collected on 300 mesh thin-bar Cu grids (EMS Hatfield, PA), and post-stained with uranyl acetate and lead citrate. The sectioned samples were viewed at 80 kV on a Philips CM120 transmission electron microscope and documented with an AMT BioSprint12 digital camera (Advanced Microscopy Techniques, Woburn, MA).

### Live cell imaging

For all live cell imaging experiments, an ER inducible release system was used such that transfected MEFs expressed a fluorescently labeled protein to an aggregation domain that trapped the protein in the ER. Following a 48 h incubation period post transfection, cells were treated with a solubilizer to induce ER release. Solubilizing agents used were dependent on construct identity and are detailed in subsequent sections. To visualize early protein trafficking events from the ER, MEFs were transfected with the 4×F_M_-mCh-NL1 plasmid (Gift from Matthew Kennedy). Before imaging, cells were treated with DDS (635,054, Takara) at a final concentration of 1 μM to induce release from the ER. Images were collected every 3 s for a duration of 3 min within 10 min of DDS induced solubilization with an A1R-HD confocal microscope (Nikon) using a 60 × water objective. Imaris imaging software with the Spots module was used to track vesicle movement. The maximum velocity of each cargo was tracked, and the mean of maximum velocity was recorded for each cell. If cargo trafficked more than 2 μm in 3 min, the cargo was categorized as “released”. The number of released cargo over total cargo in each cell was used to calculate percent release. To visualize trafficking from the ER to the Golgi, MEFs were transfected with the DsRedExpress2-FKBP(LV)-GalNAcT2-msGFP2 plasmid. As this aggregation domain is slightly different from the one used to measure ER budding, a different solubilizer, SLF (10007974, Cayman Chemical Co.), was added at a final concentration of 100 μM. Images were acquired with an ImageXpress Micro 4 (Molecular Devices) using a 40 × air objective every 2 min for a total of 30 min following solubilizer addition. Imaris imaging software was used to measure the colocalization of the green and red channels. Accumulation in the Golgi was quantified by measuring the sum of DsRed intensity as it overlapped with the GFP channel. To overcome cell-to-cell intensity variability, each cell was normalized to its maximum intensity over the 30 min. To visualize trafficking to the cell surface, MEFs were transfected with a plasmid containing 4×F_M_-Halo-L1CAM. DDS was added to cells at 1 μM. Ten minutes prior to imaging, a cell impermeant halo dye was added to the cells at a final concentration of 100 nM. Cells were then washed three times with DMEM before imaging. DDS was added to cells at 1 μM. Full cell z-stack images were taken with a TI2 Spinning Disk Confocal Microscope (Nikon) using a 60 × oil objective every 10 min from 20 to 70 min post solubilizer addition. Fluorescence intensity was measured from max intensity projections using FIJI.

### Real-time PCR

Real-time PCR (qPCR) was performed using the Roche 480 lightcycler and Sybr Green Real Time PCR Master Mix (04707416001, Roche). Expression levels were normalized against GAPDH levels, and are expressed as percent of control. PCR primers specific to each gene are summarized in Supplementary Table S2 (Bio-Rad Laboratories; Prime PCR assay). The cycling parameters were as follows: 95 °C, 10 s; 60 °C, 20 s; and 72 °C, 30 s, for a total of 45 cycles. Primers are listed in Supplementary Table S2.

### Statistics

Data analysis was performed in Graphpad Prism v 8.3.1 (GraphPad Software, Inc) and R v3.5.1. Data are expressed as mean ± standard deviation or SEM, as noted in each instance. For the proteomics, fold changes were computed within each DiLeu batch experiment. An F-test was used to test for equivalent variance among groups, and a Student’s t test was performed assuming equal or unequal variance according to the results of the F-test. A final fold change was calculated by averaging the two experiments together, and the p-values of the two separate DiLeu experiments were combined using Fisher’s method as implemented in the R package metap (R version 3.5.1). For the glycoproteomics, a Student’s t-test was performed. For trafficking experiments, a two-way ANOVA was used with multiple comparisons reported at each time point. For all other analyses, comparison of the means was performed using a Student’s t-test, or a one-way ANOVA. The following statistical significance was used: *P < 0.05; **P < 0.005; #P < 0.0005.

## Supplementary Information

Supplementary Information

## Data Availability

The proteomics data have been deposited in the ProteomeXchange Consortium via the PRIDE partner repository with the accession code PXD013736. The acetyl-proteomics data have been deposited to the ProteomeXchange Consortium via the MassIVE partner repository with the accession code PXD014013. The R script that was used to process the acetyl-proteomics data has been deposited on Github with the identifier (search terms: AT1 Acetylation Stoich) (https://doi.org/10.5281/zenodo.3238525). The N-glycoproteomics data have been depositied to the Proteome Xchange Consortium via the PRIDE partner repository with the data set identifier PXD019770. The authors declare that all other data supporting the findings of this study are available within the paper and its supplementary tables.
