# Supplementary material for: Acetyl-CoA flux from the cytosol to the ER regulates engagement and quality of the secretory pathway
Source: Sci Rep. 2021 Jan 21;11:2013. doi: 10.1038/s41598-021-81447-6 (PMC7820588; doi:10.1038/s41598-021-81447-6)
Supplement: Supplementary file 1 — Supplementary Information [file 41598_2021_81447_MOESM1_ESM.pdf]

SUPPLEMENTARY MATERIAL

Acetyl-CoA flux from the cytosol to the ER regulates engagement and quality of the secretory pathway

Inca A. Dieterich, Yusi Cui, Megan M. Braun, Alexis J. Lawton, Nicklaus H. Robinson, Jennifer Peotter, Qing Yu, Jason C. Casler, Benjamin S. Glick, Anjon Audhya, John M. Denu, Lingjun Li, Luigi Puglielli

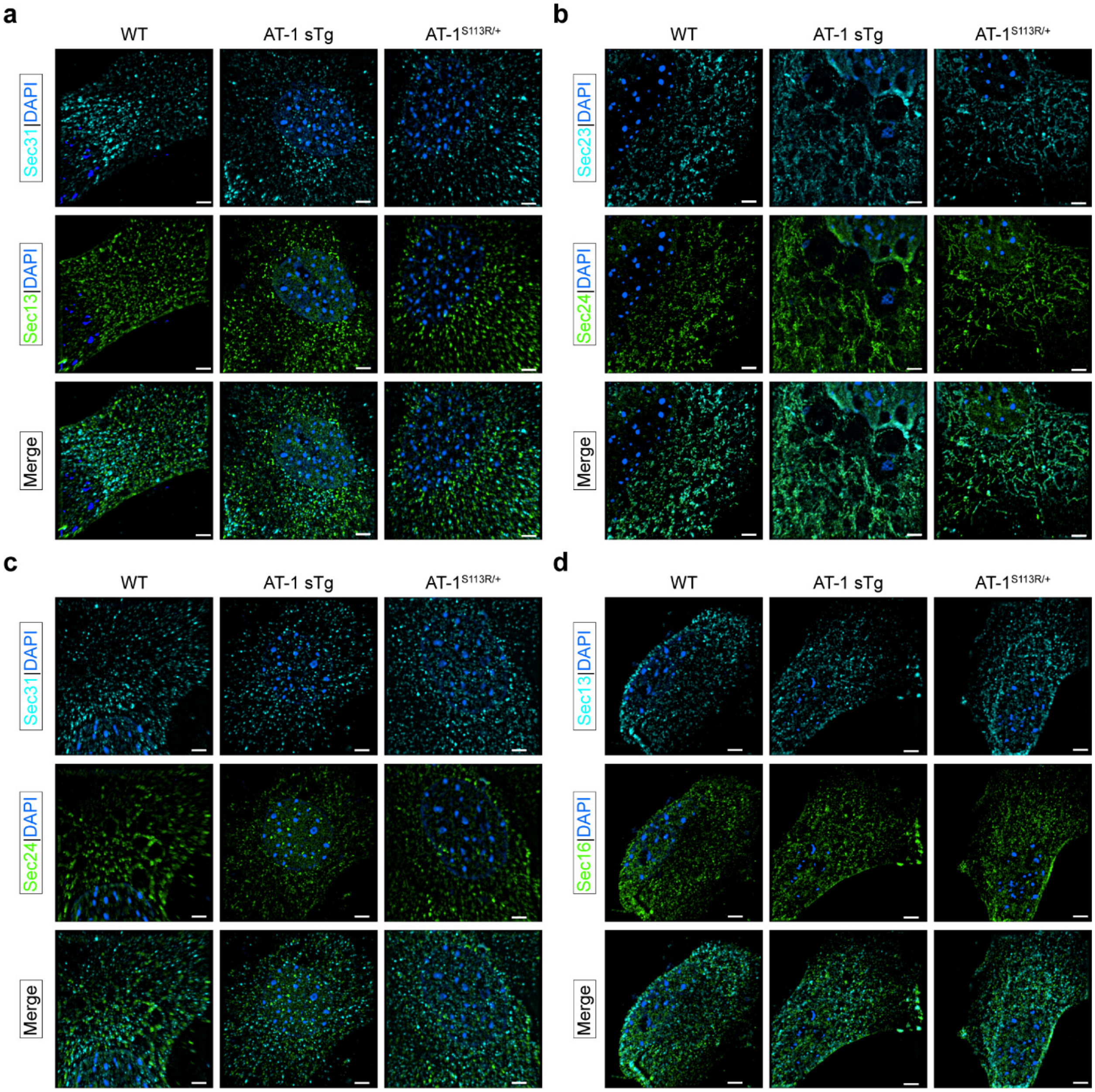

Supplementary Figure S1

Supplementary Figure S1. Aberrant AT-1 models show no major changes in number nor assembly of COPII cargo structures.

Representative SIM images of COPII proteins in primary-cultured MEFs: (**a**) Sec31 and Sec13; (**b**) Sec23 and Sec24; (**c**) Sec 31 and Sec24; (**d**) Sec13 and Sec16. Scale bar, 3 µm.

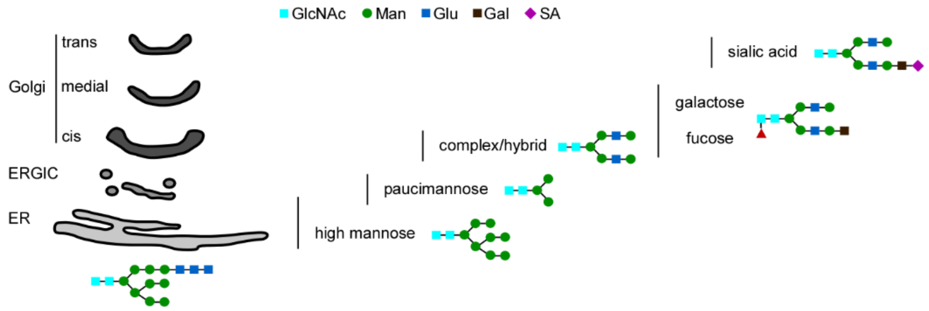

Supplementary Figure S2

Supplementary Figure S2. Schematic view of N-glycan modification across the secretory pathway.

The preformed ER-based $GlcNAc_2Man_9Glu_3$ is processed as the nascent glycoprotein transits through the Golgi apparatus. Only general oligosaccharide structures are shown here. Simple and complex variations of the structure presented here are possible.

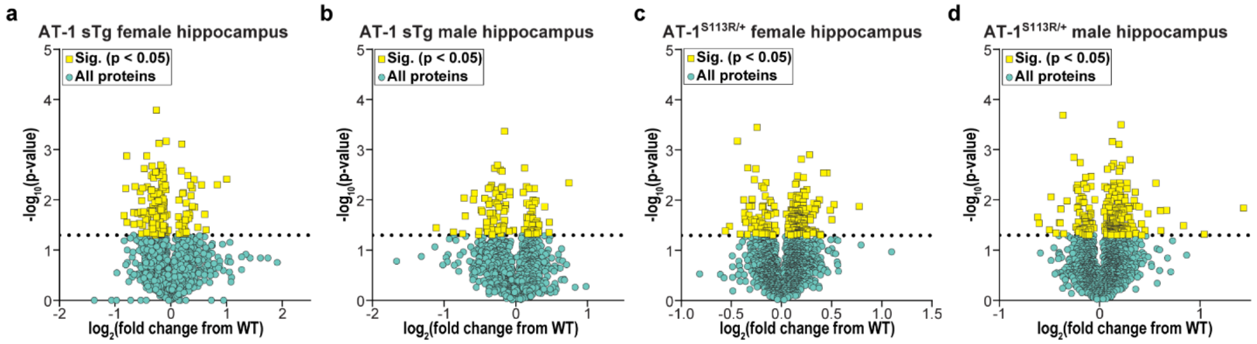

e

AT-1 sTg female hippocampus

Glycan Type	Example	Identified glycans (sig./**total**)
Sialylated		57/**1338**
Fucosylated		66/**1146**
Complex/hybrid		13/**297**
Paucimannose		0/**67**
High mannose		44/**870**

f

AT-1 sTg male hippocampus

Glycan Type	Example	Identified glycans (sig./**total**)
Sialylated		44/**1338**
Fucosylated		33/**1146**
Complex/hybrid		8/**297**
Paucimannose		4/**67**
High mannose		22/**870**

g

AT-1$^{S113R/+}$ female hippocampus

Glycan Type	Example	Identified glycans (sig./**total**)
Sialylated		49/**1336**
Fucosylated		77/**1146**
Complex/hybrid		17/**267**
Paucimannose		8/**82**
High mannose		28/**887**

h

AT-1$^{S113R/+}$ male hippocampus

Glycan Type	Example	Identified glycans (sig./**total**)
Sialylated		60/**1336**
Fucosylated		75/**1146**
Complex/hybrid		18/**267**
Paucimannose		5/**82**
High mannose		56/**887**

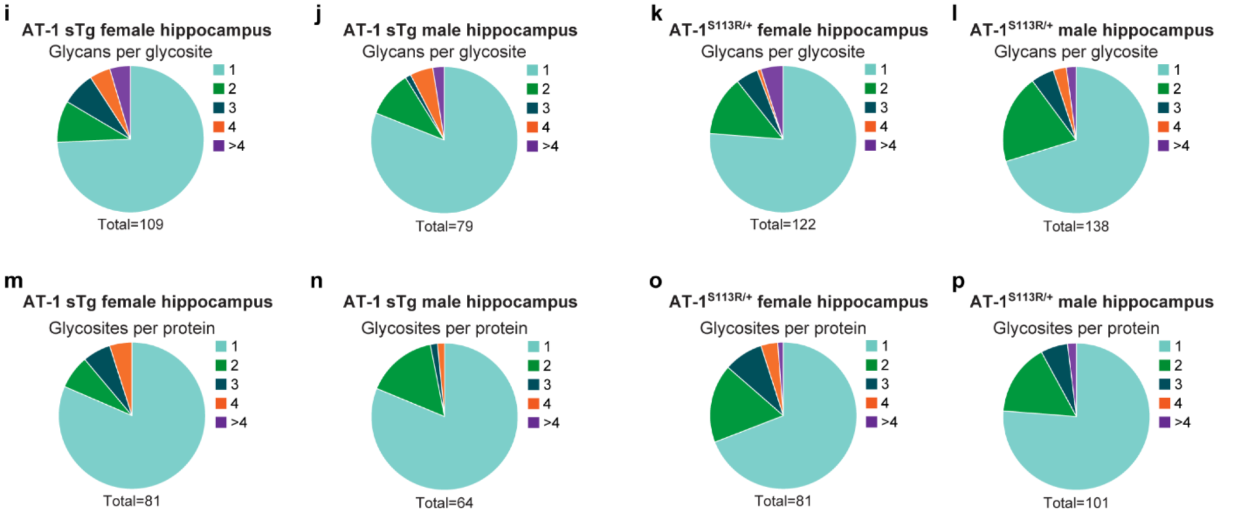

Supplementary Figure S3

Supplementary Figure S3. AT-1 sTg and AT-$1^{S113R/+}$ display N-glycoproteomic changes across many hippocampal glycoforms.

a, b, Volcano plot displaying all quantified glycoproteins in the hippocampus of AT-1 sTg (**a**) female (n = 3) and (**b**) male (n = 3), compared with age-matched WT littermates. Statistically significant proteins (180 in female; 112 in male) are highlighted in yellow, and all other proteins are designated in blue. Student's T-test, $P < 0.05$.

c, d, Volcano plot displaying all quantified glycoproteins in the hippocampus of AT-$1^{S113R/+}$ (**c**) female (n = 3) and (**d**) male (n = 3), compared with age-matched WT littermates. Statistically significant proteins (179 in female; 214 in male) are highlighted in yellow, and all other proteins are designated in blue. Student's T-test, $P < 0.05$.

e, f, g, h, Identified glycans are categorized into five glycan types, and are divided by significant over total identified in hippocampal (**e**) AT-1 sTg female, (**f**) AT-1 sTg male, (**g**) AT-$1^{S113R/+}$ female, and (**h**) AT-$1^{S113R/+}$ male.

i, j, k, l, Significant glycans per glycosite are identified in hippocampal (**i**) AT-1 sTg female, (**j**) AT-1 sTg male, (**k**) AT-$1^{S113R/+}$ female, and (**l**) AT-$1^{S113R/+}$ male.

m, n, o, p, Significant glycosites per protein are identified in hippocampal (**m**) AT-1 sTg female, (**n**) AT-1 sTg male, (**o**) AT-$1^{S113R/+}$ female, and (**p**) AT-$1^{S113R/+}$ male.

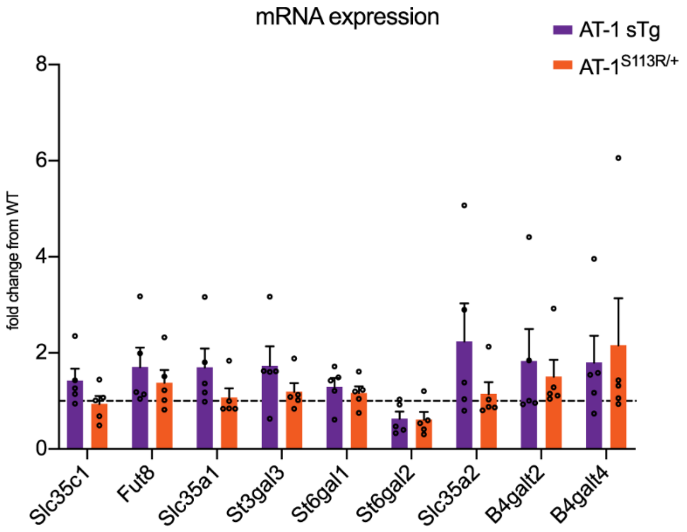

Supplementary Figure S4

Supplementary Figure S4. AT-1 sTg and AT-1$^{S113R/+}$ show no difference in mRNA of Golgi-specific glycosylation proteins.

a, mRNA levels of *Slc35c1*, *Fut8*, *Slc35a1*, *St3gal3*, *St6gal1*, *St6gal2*, *Slc35a2*, *B4galt2*, and *B4galt4* in MEF samples (MEFs from biologically independent animals; WT, n = 5; AT-1 sTg, n = 5; AT-1S113R/+, n = 5). Two-tailed Student's t test.

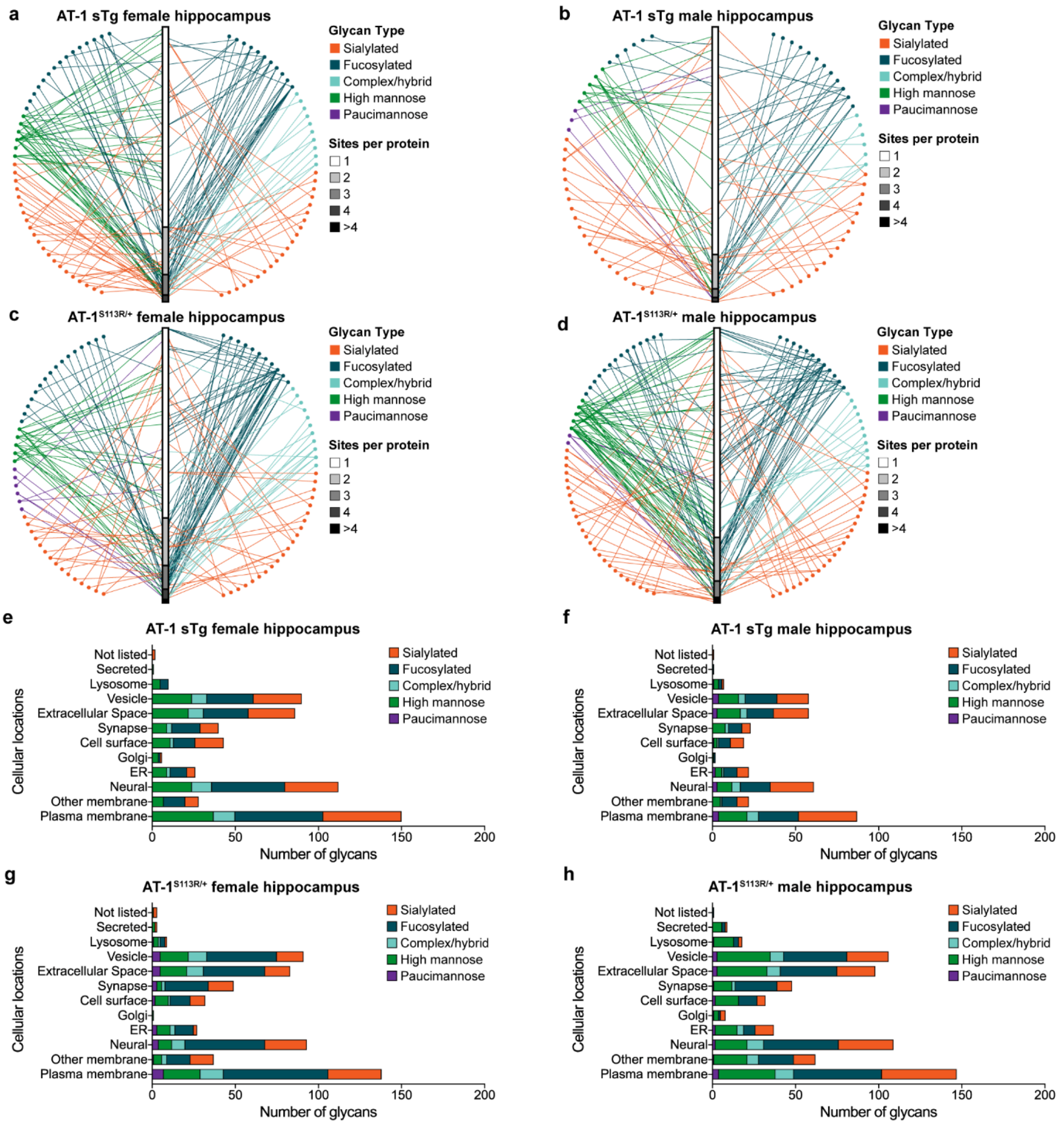

Supplementary Figure S5

Supplementary Figure S5. AT-1 sTg and AT-1$^{S113R/+}$ display a highly heterogeneous hippocampal secretome.

a, b, c, d Network of glycan type plotted to glycoprotein for hippocampal (**a**) AT-1 sTg female, (**b**) AT-1 sTg male, (**c**) AT-1$^{S113R/+}$ female, and (**d**) AT-1$^{S113R/+}$ male. Circle represents glycan type, with each node representing a specific glycan tree. Glycans intersect with their protein, organized by the number of glycosites identified on that glycoprotein.

e, f, g, h, Glycosylation distribution according to subcellular localizations as defined by GO cellular component terms in hippocampal (**e**) AT-1 sTg female, (**f**) AT-1 sTg male, (**g**) AT-1$^{S113R/+}$ female, and (**h**) AT-1$^{S113R/+}$ male.

Supplementary Table S1. Cluster analysis of secretory pathway-related proteins (from Fig. 1)

	AT-1 sTg		AT-1$^{S113R/+}$	
Gene symbol	proteome	acetyl-proteome site (change)	proteome	acetyl-proteome site (change)
Lman1	0.313	ns	0.081; 0.026	ns
Sec31a	ns	K200 (0.470)	ns	K200 (-0.293)
Sec13	ns	ns	-0.367	ns
Hspa1l	0.239	ns	ns	ns
Prkcsh	0.500	ns	-0.529	K206 (0.214)
Cyfip1	-0.976	ns	ns	ns
Pdia3	ns	K129 (-0.799); K173 (0.002); K448 (-0.093); K75 (0.063)	ns	K271 (0.113)
Ero1lb	-1.126	ns	ns	ns
Ero1l	0.346; -0.494	ns	ns	ns
Pabpc1	-0.526	K30 (-0.037); K333 (-0.089)	-0.560	K229 (0.028); K231 (0.002)
Nup133	-0.219	ns	ns	ns
Phax	-0.753; -1.042	ns	ns	ns
Nup37	-0.496	ns	ns	ns
Dnaja1	ns	K14 (-0.009)	ns	ns
Hsp90ab1	0.276	K69 (-0.355)	ns	K435 (0.606); K607 (0.018)
Calr	0.597	K351 (-0.197)	ns	ns
P4hb	0.599; 0.596; 0.502	K426 (-0.207; 0.047); K446 (-0.011)	ns	K33 (-0.146)
Usp39	-0.035	ns	ns	ns
Rbm25	0.566; -0.642	ns	ns	ns
Nup188	-0.413	ns	ns	ns
Ranbp2	0.826	ns	ns	ns
Nup54	0.423	ns	ns	ns
Hsp90b1	ns	K75 (-0.237)	ns	K168 (0.081)
Rpn2	0.801	ns	ns	ns
Sel1l	0.530	ns	ns	ns
Vimp	0.725; 0.770	ns	ns	ns
Sart1	0.743; -1.008	ns	ns	ns
Pqbp1	-0.637	ns	ns	ns

Srsf5	-0.560	ns	ns	ns
Nup155	-0.276	ns	ns	ns
Hspa5	ns	K327 (0.116); K634 (0.102)	ns	K634 (-0.148); K341 (0.589); K475 (0.271)
Hsp90aa1	-0.561	K447 (-0.620); K500 (0.082)	ns	ns
Os9	0.382	ns	ns	ns
Vcp	ns	K651 (-0.012)	ns	ns
Ssr4	0.790	ns	ns	ns
Hnrnpm	0.348; 0.350	ns	ns	ns
Sf3b5	-0.008	ns	ns	ns
Ddx42	-0.816	ns	ns	ns
Hspa1b	-0.672	K220 (-0.201)	ns	K220 (0.336)
Dnajc3	0.533; 0.042	ns	ns	ns
Ddost	0.479	ns	ns	ns
Plaa	-0.285	ns	ns	ns
Dhx8	-0.629	ns	ns	ns
Pcbp1	0.555	ns	-0.335	K314 (-0.153)
Ddx46	0.517	ns	ns	ns
Hspa8	ns	ns	-0.189	K500 (-0.102)
Hspa1a	-0.672	ns	ns	ns
Sec63	0.288; -0.356	ns	ns	ns
Psmd1	0.370	ns	ns	ns
Nploc4	-0.487	ns	ns	K307 (0.126)
Prpf8	-0.361	ns	ns	ns
Sf3a3	-0.768	ns	-0.343	ns
Phf5a	0.910	ns	ns	ns
Cdc5l	-1.098	ns	ns	ns
Sec62	-0.596	ns	ns	ns
Uba52	0.857	ns	ns	ns
Psmd11	0.681	ns	ns	ns
Psma5	0.426; 0.501	ns	ns	ns
Psma7	0.736; 0.718; 0.723	ns	ns	ns
Sf3b4	-0.498	ns	ns	ns
Magoh	-1.519	ns	ns	ns
Eif2ak2	-0.467	ns	ns	ns

Rpl18a	0.744	ns	ns	ns
Rpl14	1.143	ns	ns	ns
Psmc6	0.438	ns	ns	ns
Psmd13	0.320	ns	ns	ns
Psma2	0.672	ns	ns	ns
Psma1	0.466; 0.690; 0.692	ns	ns	ns
Rps2	ns	ns	-0.175; -0.176	ns
Rpl13	0.500; 0.462	ns	ns	ns
Rpl27a	1.769	ns	ns	ns
Sec61a1	0.576; 0.574	ns	ns	ns
Psmd6	-0.287	ns	ns	ns
Psmb1	-0.396	ns	-0.082	ns
Rps27l	-0.786	ns	ns	ns
Rplp0	0.387; 0.476; 0.491	ns	ns	ns
Rplp2	0.552	ns	ns	ns
Sec61b	0.828	ns	ns	ns
Psma6	0.631	ns	ns	ns
Psme4	-1.092	K36 (0.051)	ns	ns
Eif4a2	-0.785	ns	-0.291	ns
Eif2s1	0.268	ns	ns	ns
Rps18	0.045	K25 (0.107)	ns	ns
Rps25	0.202	ns	ns	ns
Rpl19	0.003	K46 (-0.034)	ns	ns
Rpl6	0.344	ns	0.289	ns
Rpl26	0.658	K2 (0.061)	ns	ns
Rad23a	-0.320	ns	ns	ns
Eif4b	-0.694	ns	ns	ns
Eif3d	0.198	ns	ns	K138 (-0.002)
Rps23	0.907	ns	ns	ns
Rps15a	ns	ns	ns	K71 (-0.192)
Rpl3	0.321; 0.483	ns	ns	ns
Rpl8	0.868	K177 (0.004); K234 (-0.321)	ns	ns
Ube2d3	-0.343	ns	ns	ns
Eif5	0.471	ns	ns	ns

Eif3j1	-0.403	ns	ns	ns
Rps9	0.538	K139 (-0.056)	ns	K47 (-0.057)
Rps19	0.414	ns	ns	ns
Rps21	0.923	ns	ns	ns
Rpl18	ns	K78 (-0.246)	ns	ns
Rpl28	ns	K22 (0.119); K72 (-0.366)	ns	K72 (0.296); K22 (0.113)
Ube2j2	-0.553	ns	ns	ns
Eif3j2	-0.403	ns	ns	ns
Rps28	0.398	ns	-0.296	ns
Rps16	0.496	ns	ns	ns
Rps17	0.922; 0.658	ns	ns	K72 (0.055)
Rpl5	0.598; 0.683	ns	ns	K270 (0.025); K197 (0.112)
Rpl35	1.028	K35 (-0.070)	ns	ns
Eif3b	0.426	ns	ns	ns
Rps20	1.727; 0.505	ns	ns	ns
Mrpl15	-0.355	ns	ns	ns
Rpsa	-0.270	ns	-0.260; -0.415	ns
Mrpl24	-0.035	ns	ns	ns
Rpl13a	0.395; 0.817	ns	ns	K51 (0.243)
Mrpl10	-0.253	ns	ns	ns
Mrpl18	0.181	ns	ns	ns

Supplementary Table S2. Primers used for Real Time PCR.

**Primer Pair**	**Forward Sequence**	**Reverse Sequence**
SLC35C1	5'-GCGCTGACTGGAGTCTCTG-3'	5'-AGGTGACAAAAATGGGGGTATC-3'
FUT8	5'-CTTGCAGACCGACTCCTAAGA-3'	5'-CAAGGTTGTGGACGAATCAAGT-3'
SLC35A2	5'-GTTGGAACCTGGGTCCACTAC-3'	5'-GGTGAGACCTTTGAGCACTTC -3'
B4GALT2	5'-GGAGCACCACCTACGCTATTG-3'	5'-GATTCGGGTCATCCATAGGGA-3'
B4GALT4	5'-GCCATCCTCATTCCACAC-3'	5'-GTCCCAGTTCTCCTCCTT-3'
B4GALT5	5'-CCAACCACGACCTTTCTTCCT-3'	5'-ATCGTCCATTGCGATCTCACT-3'
SLC35A1	5'-GCTCCGGCGAGAGAAAATGT-3'	5'-TCTTAAAGCTACGGTGTAAGCG-3'
ST3GAL1	5'-AAGCTGGACTCTAAACTGCCT-3'	5'-TGCTGGCTTGGAGAACCTG-3'
ST6GAL1	5'-CTCCTGTTTGCCATCATCTGC-3'	5'-GGGTCTTGTTTGCTGTTTGAGA-3'
ST6GAL2	5'-CCAGGTGTACCTCTGAGCCA-3'	5'-AGCACTGTCAATGAAGTGATGG-3'
GAPDH	5'-AGGTCGGTGTGAACGGATTTG-3'	5'-TGTAGACCATGTAGTTGAGGTCA-3'
